# Supplementary material for: Identifying promoter sequence architectures via a chunking-based algorithm using non-negative matrix factorisation
Source: PLoS Comput Biol. 2023 Nov 20;19(11):e1011491. doi: 10.1371/journal.pcbi.1011491 (PMC10695386; doi:10.1371/journal.pcbi.1011491)
Supplement: S1 Text — (PDF) [file pcbi.1011491.s001.pdf]

Supplementary for 'Identifying promoter sequence architectures via a chunking-based algorithm using non-negative matrix factorisation'

Sarvesh Nikumbh[1,2,*] Boris Lenhard[1,2,*]

Contents

1 Supplementary figures from experiments on simulated data 2

2 Results on Drosophila core promoters from modENCODE 3

3 seqArchR parameters choices for all organisms 4

4 Supplementary figures of results on Drosophila core promoters from Schor et al.[3] 6

4.1 Visualisation showing curation of seqArchR result clusters for D. melanogaster 6

4.2 Cluster containing non-His2B genes with His2B genes 10

4.3 Proportions of genomic annotations for promoters for D. melanogaster 12

4.4 seqArchR results for D. melanogaster using stability bound 10^{-8} 12

4.5 Ensuring identification of TATA-box at all stages in D. melanogaster development 16

4.6 GO terms enriched for different clusters in Drosophila melanogaster development 27

5 Supplementary figures of results on Zebrafish core promoters from [6] 29

5.1 Visualisation showing curation of seqArchR result clusters for D.rerio 29

5.2 Motif heatmaps for Zebrafish development stages 32

5.3 Chromosomal locations of promoters/CTSSs in seqArchR clusters for Zebrafish development stages . 35

5.4 Overlaps between promoter sequences at all stages in D.rerio 39

5.5 Top-10 GO term enrichments for clusters 40

6 Supplementary figures of results on human cell lines core promoters from ENCODE 42

6.1 Visualisation showing curation of seqArchR result clusters for H.sapiens 42

6.2 Comparison of cluster architectures in shorter vs longer downstream flank scenario 44

6.3 Comparison of cluster architectures in two scenarios: with *vs* without initiator sequence 46

6.4 Diminished tissue-specific signal among the all-pooled CAGE data for H. sapiens 48

References 49

[1] MRC London Institute of Medical Sciences, London, UK
[2] Institute of Clinical Sciences, Faculty of Medicine, Imperial College London, Hammersmith Hospital Campus, London, UK

[*] Correspondence: Sarvesh Nikumbh <s.nikumbh@imperial.ac.uk>, Boris Lenhard <b.lenhard.imperial.ac.uk>

1 Supplementary figures from experiments on simulated data

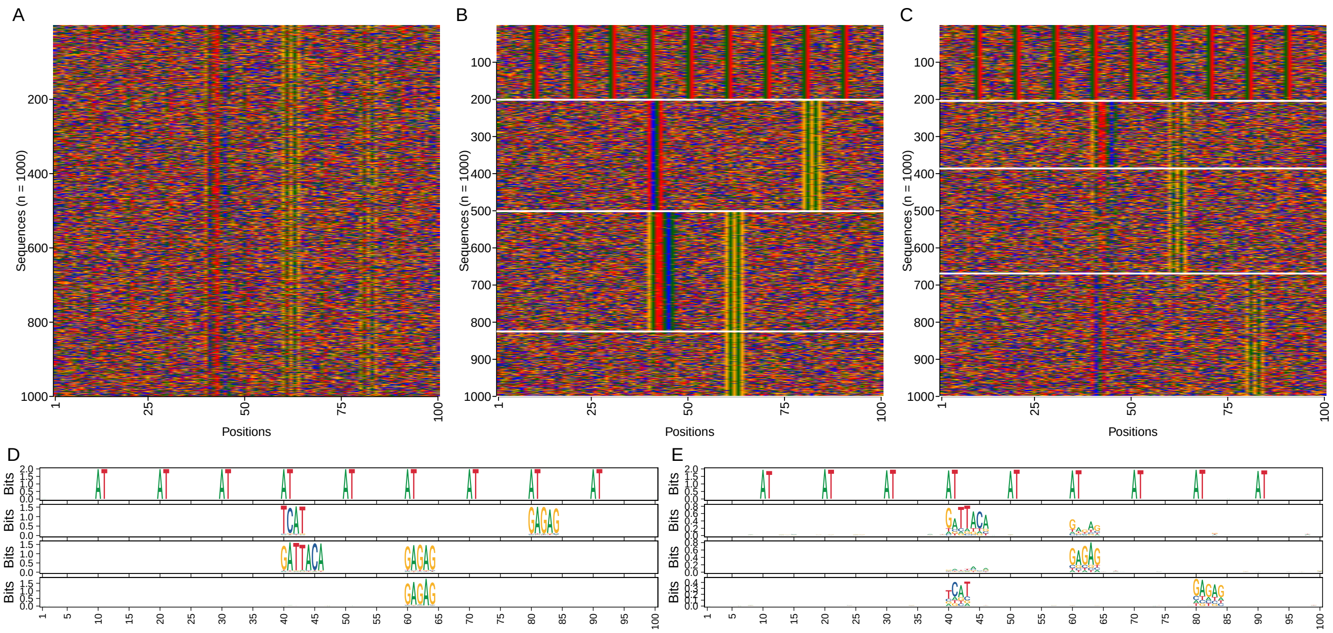

Fig A. Qualitative assessment of result on simulated data. (**A**) Sequences visualised as an image for randomised input, (**B, C**) clustered output and sequence logos from seqArchR for $m = 0.1$ and $p = 1$, and (**D, E**) for $m = 0.5$ and $p = 3$.

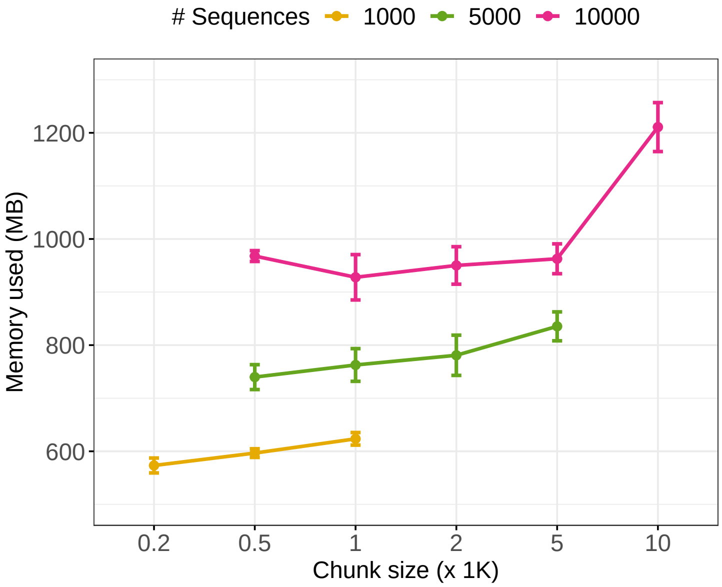

Fig B. Memory footprint of seqArchR. The maximum resident set sise for different settings of seqArchR is reported.

2 Results on Drosophila core promoters from modENCODE

We processed CAGE-derived core promoter sequences for Drosophila melanogaster available from modENCODE [1] with seqArchR to facilitate comparison with NPLB [2] on real promoter sequences. Specifically, 6635 core promoter sequences of D. melanogaster carcass [1] were processed with seqArchR using no parallelisation. We let seqArchR perform five iterations with collation performed at only the second iteration. While NPLB takes about 600 minutes to report 12 promoter architectures in the first pass, a comparable result from seqArchR is obtained at the end of second iteration reporting seven architectures and taking 27 minutes. NPLB further processes all the architectures identified in the first pass [ˆ1] to obtain a total of 30 architectures. [ˆ1]: Each cluster of sequences is manually processed.

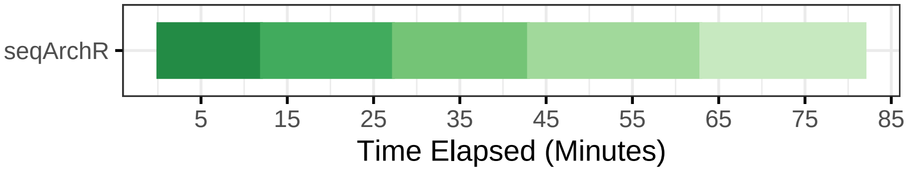

Fig C. Time taken by seqArchR to process CAGE-derived core promoter sequences from D. melanogaster (modENCODE) with no parallelisation. Each shade of green represents time taken by an individual iteration – left to right/dark to light green) iteration 1 to 5.

Figure Fig D shows the architectures/clusters identified by seqArchR at the end of iteration 2. These clusters are processed further for three additional iterations. Figure Fig E shows the cluster architectures at the end of iteration 4.

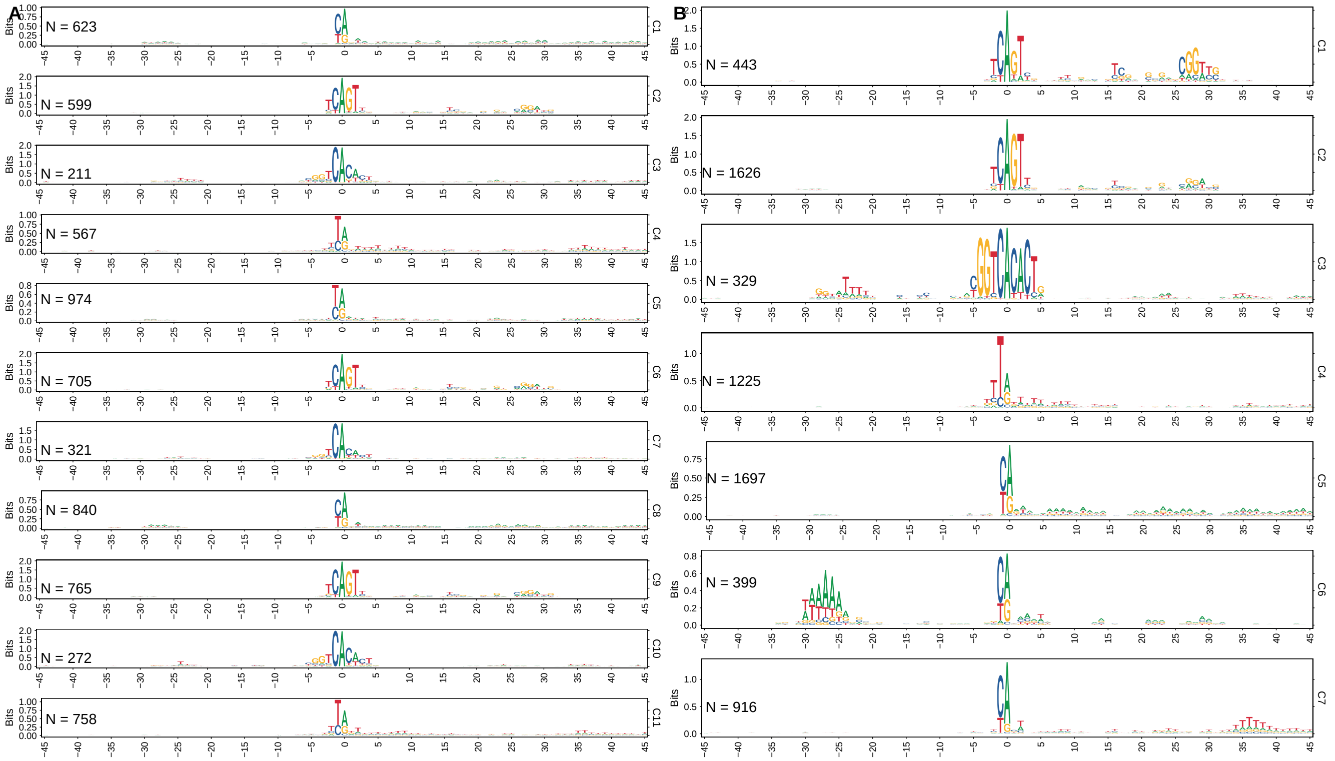

Fig D. Sequence logos of promoter architectures identified by seqArchR in D. melanogaster (modENCODE) **(A)** Iteration 1; **(B)** Iteration 2.

3 seqArchR parameters choices for all organisms

The seqArchR results reported in this work are obtained using the following parameter values. The values chosen for different parameters for seqArchR are given in Table A.

Table A. Choices for each organism for preparing data and analysing with seqArchR. minTPM, minimum Tags per million, Bound value for instability of identified clusters.

Organism	Stage	minTPM	Flank size	Chunk size	Bound value	#Iterations	Collation strategy
Fruit fly	2-4h	1	−45, +45	5000	1e-08	5	FTTTF
--'--	6-8h	1	−45, +45	5000	1e-08	5	FTTTF
--'--	10-12h	1	−45, +45	5000	1e-08	5	FTTTF
Zebrafish	64 cells	1	−45, +150	5000	1e-06	5	FTTTF
--'--	Dome	1	−45, +150	5000	1e-06	5	FTTTF
--'--	Prim-6	1	−45, +150	5000	1e-07	5	FTTTF
Human		1	−50, +5	5000	1e-06	5	FTTTF
		1	−50, +150	5000	1e-06	5	FTTTF

The collated clusters for each fruitfly stage (shown in Supplementary Figures Fig F, Fig G, and Fig H) from seqArchR were further processed using lenient bound values as reported in sub-section "Ensuring identification of TATA-box at all stages in D. melanogaster development".

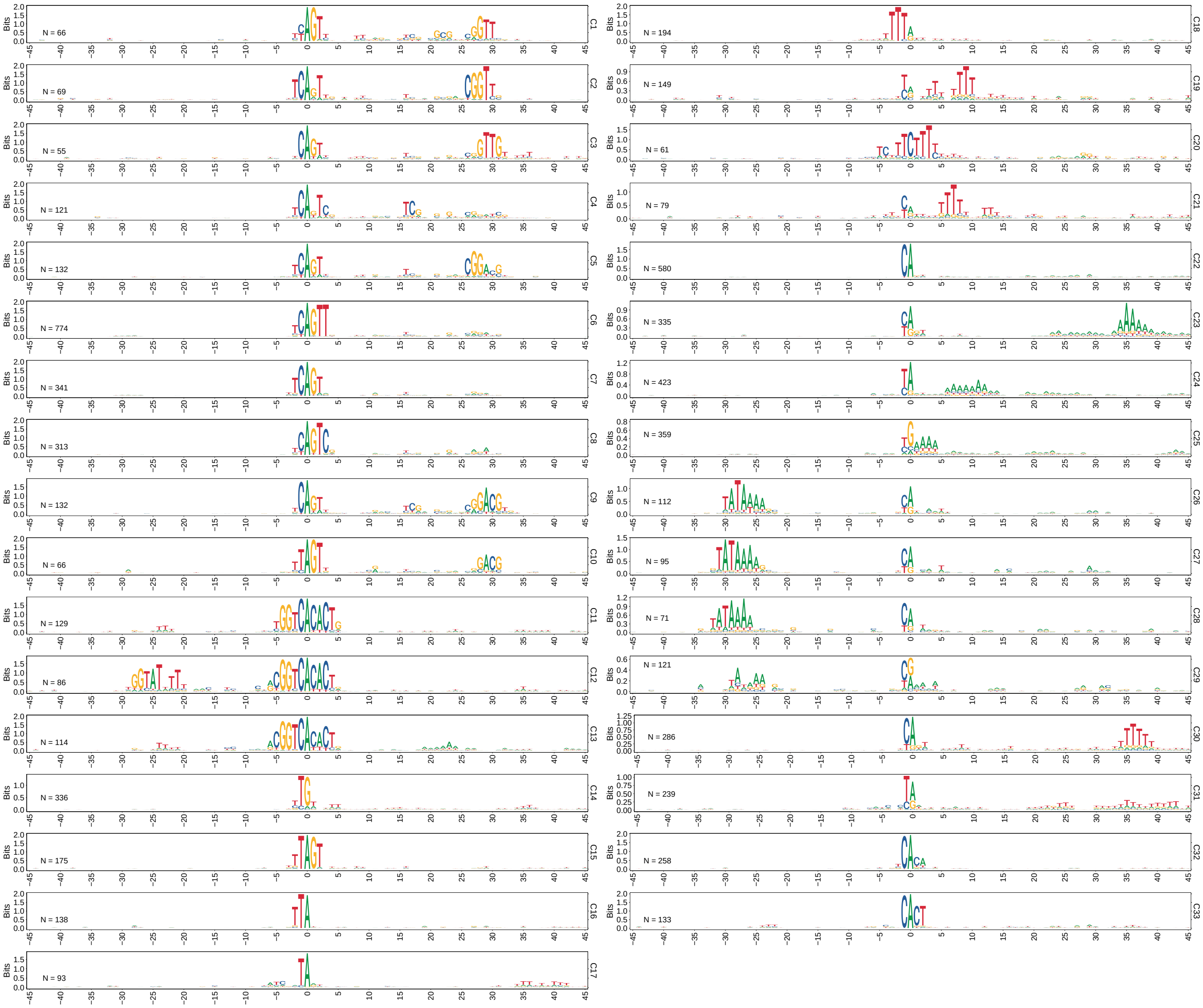

Fig E. Sequence logos of promoter architectures identified by seqArchR in D. melanogaster (modENCODE) (Iteration 4)

4 Supplementary figures of results on Drosophila core promoters from Schor et al.[3]

4.1 Visualisation showing curation of seqArchR result clusters for D. melanogaster

In Figures Fig F, Fig G, and Fig H, we show the curation of raw clusters (of promoter sequences) from seqArchR result for the different stages processed in D. melanogaster.

Note that here there are two sets of clusters arranged in two columns. The one on the left is the set of clusters identified in the fifth iteration of seqArchR. These are ordered from top to bottom by the leaves of the dendrogram (on the left) visualising the hierarchical agglomerative clustering used as part of the collation and curation step (see Methods section). The set of clusters in the right column are the curated clusters ordered by their interquantile widths (IQW).

Finally, the main text figures 4, 5 and 6 showing architectures for D. melanogaster accommodate the minor misclassifications of `TATA`-box promoters using bound value 10^{-8} but identified with lenient bound values as reported/discussed in sub-section "Ensuring identification of TATA-box at all stages in D. melanogaster development".

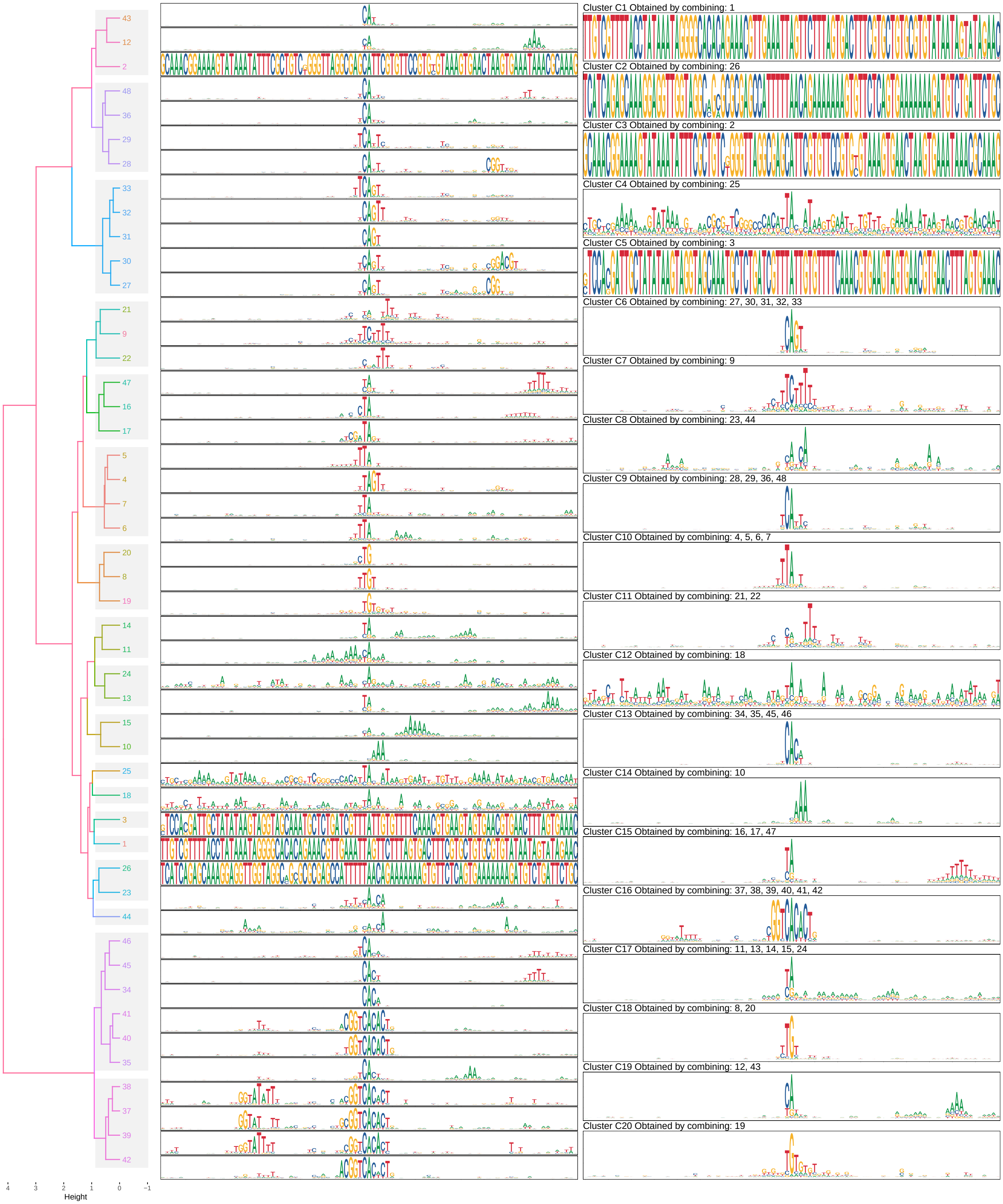

Fig F. Visualisation of the collation and curation of clusters from seqArchR raw result for 2-4h AEL, D. melanogaster

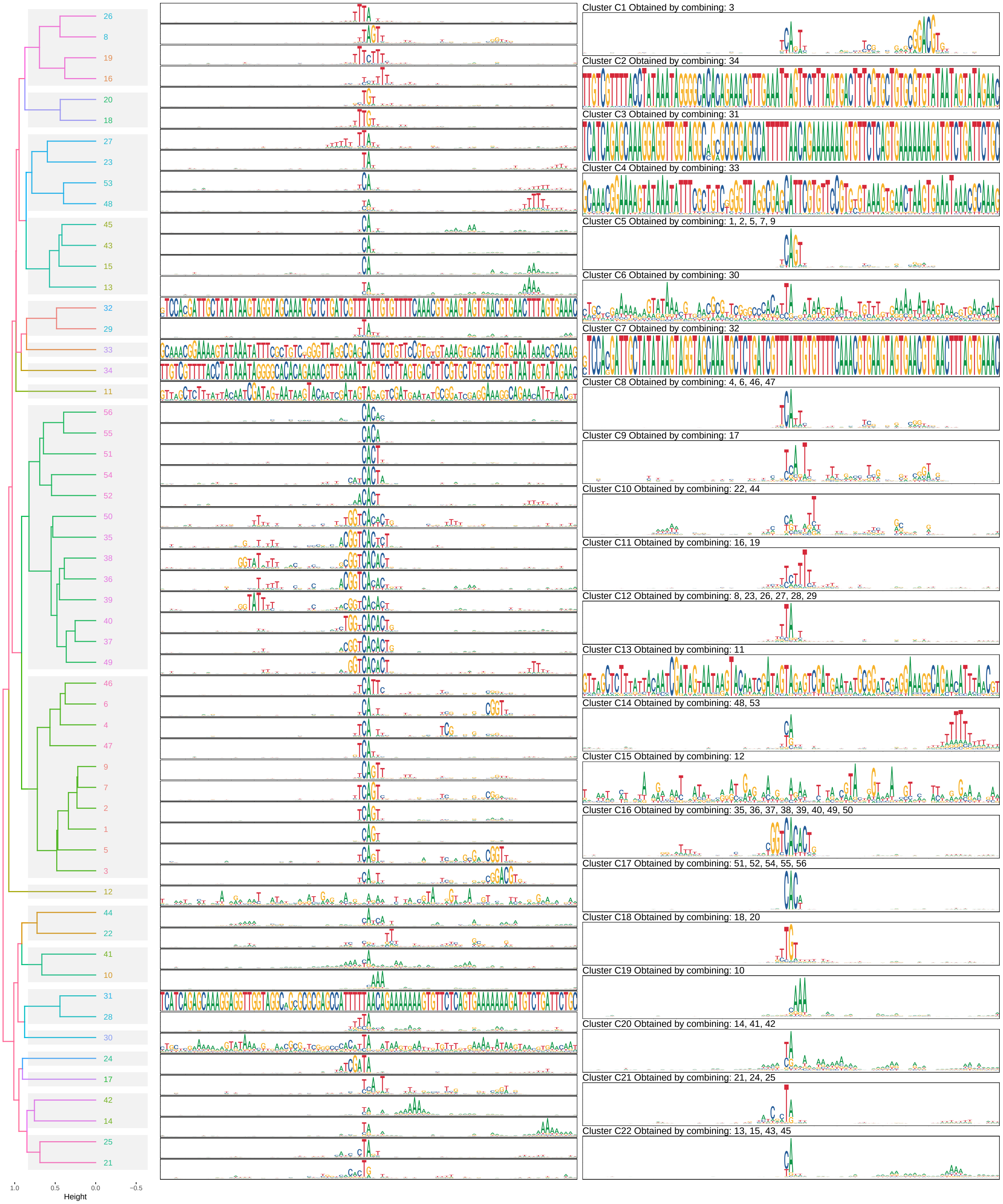

Fig G. Visualisation of the collation and curation of clusters from seqArchR raw result for 6-8h AEL, D. melanogaster

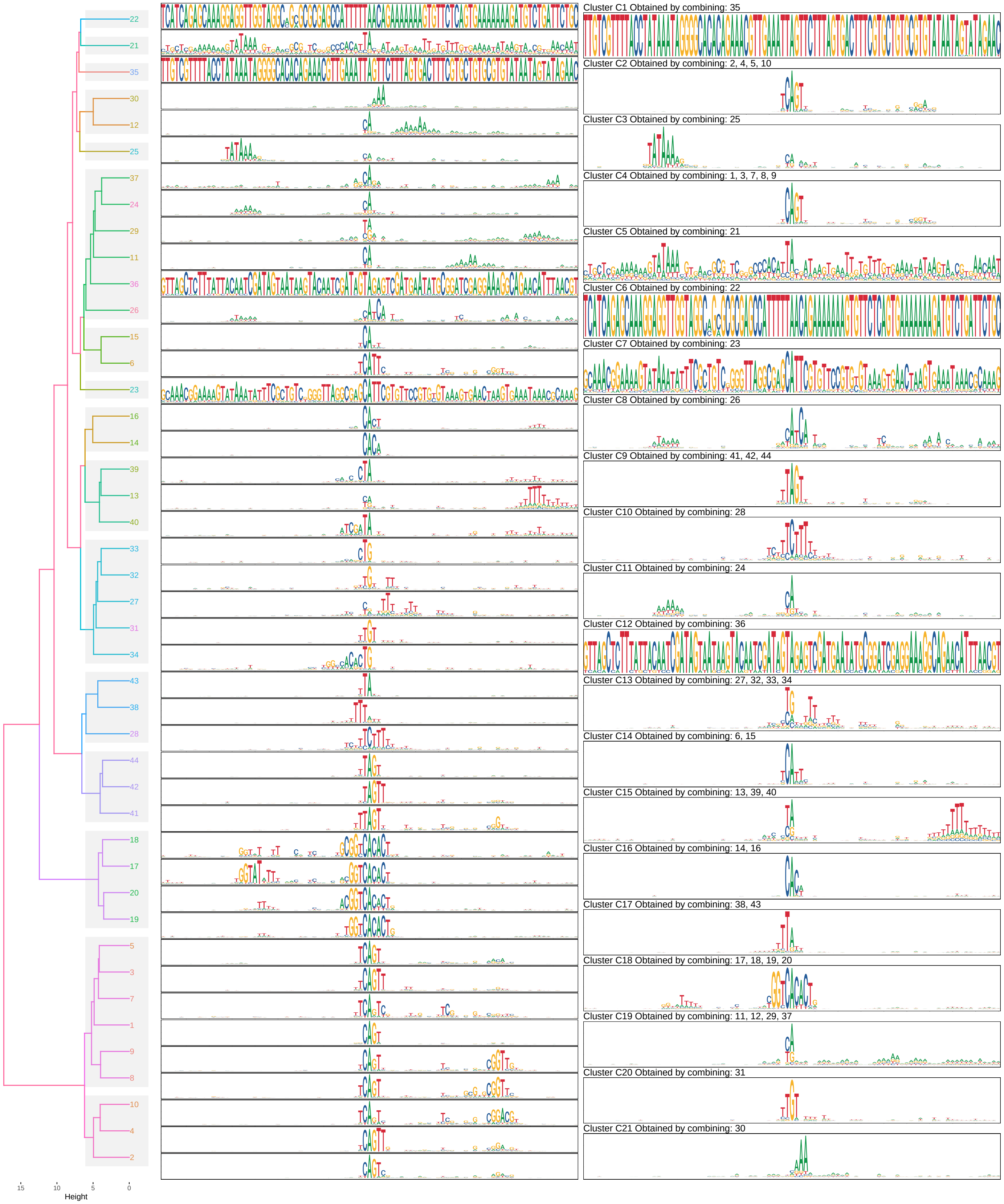

Fig H. Visualisation of the collation and curation of clusters from seqArchR raw result for 10-12h AEL, D. melanogaster

4.2 Cluster containing non-His2B genes with His2B genes

Observe that while most identified clusters with histone gene promoters are exclusive, only His2B promoters have been categorised with other non-His2B promoter sequences. We note that this is due to our approach to detect over-fitting (see step 2c in the Algorithm in the Methods section in the main text). NMF does separate out the non-His2B promoters from this cluster, but the over-fitting procedure adjudges it as overfit, and seqArchR, thus, re-assigns them back together with the base cluster (bringing His2B + non-His2B promoters together). Hence, the overall set of sequences remains unclustered/corrupted in the final result.

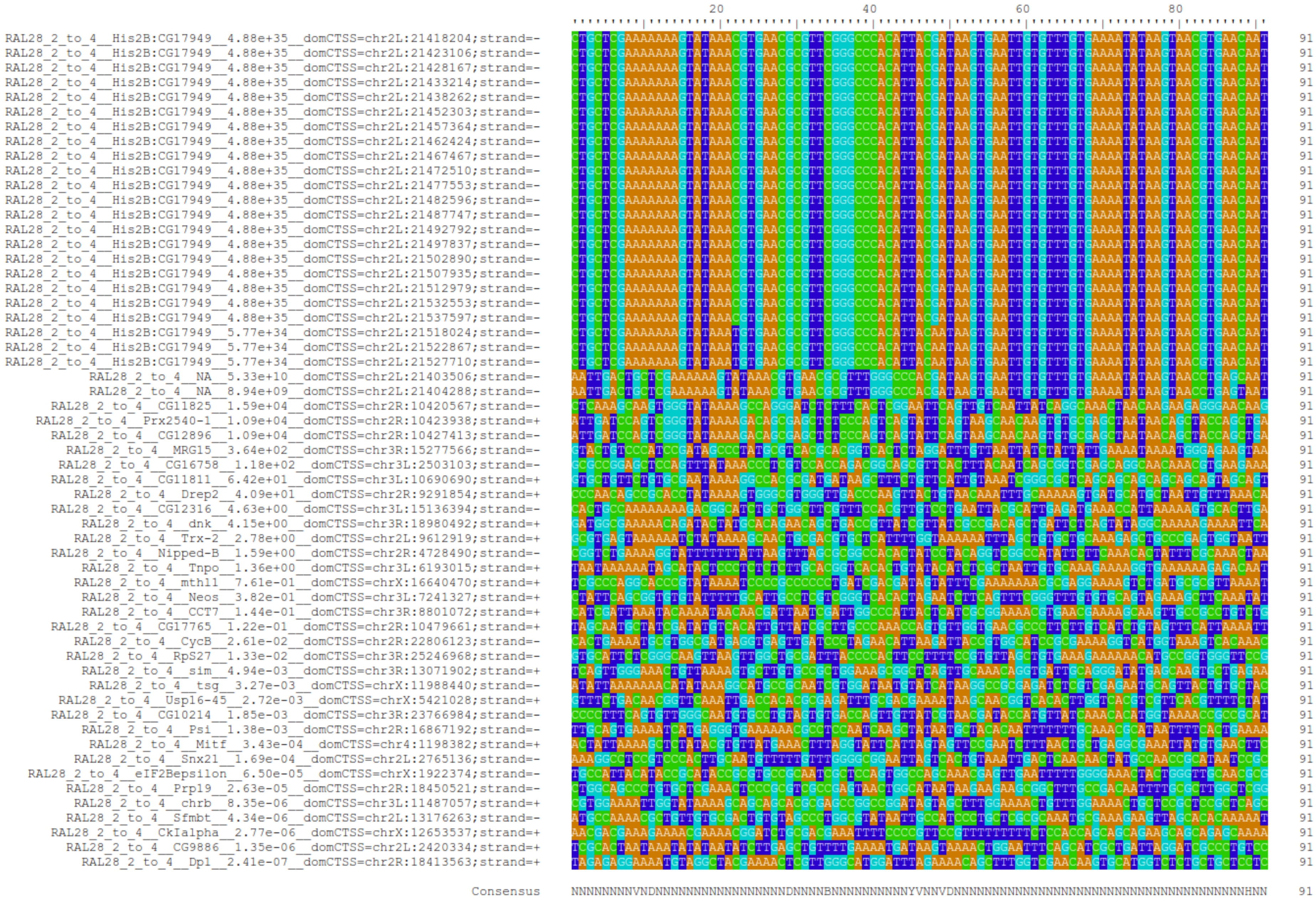

Fig I. Visualisation of alignment of sequences in cluster C4X in D. melanogaster results

4.3 Proportions of genomic annotations for promoters for D. melanogaster

Figure Fig J shows the proportion of genomic annotations for promoters in each seqArchR cluster for all three developmental stages analysed in D. melanogaster.

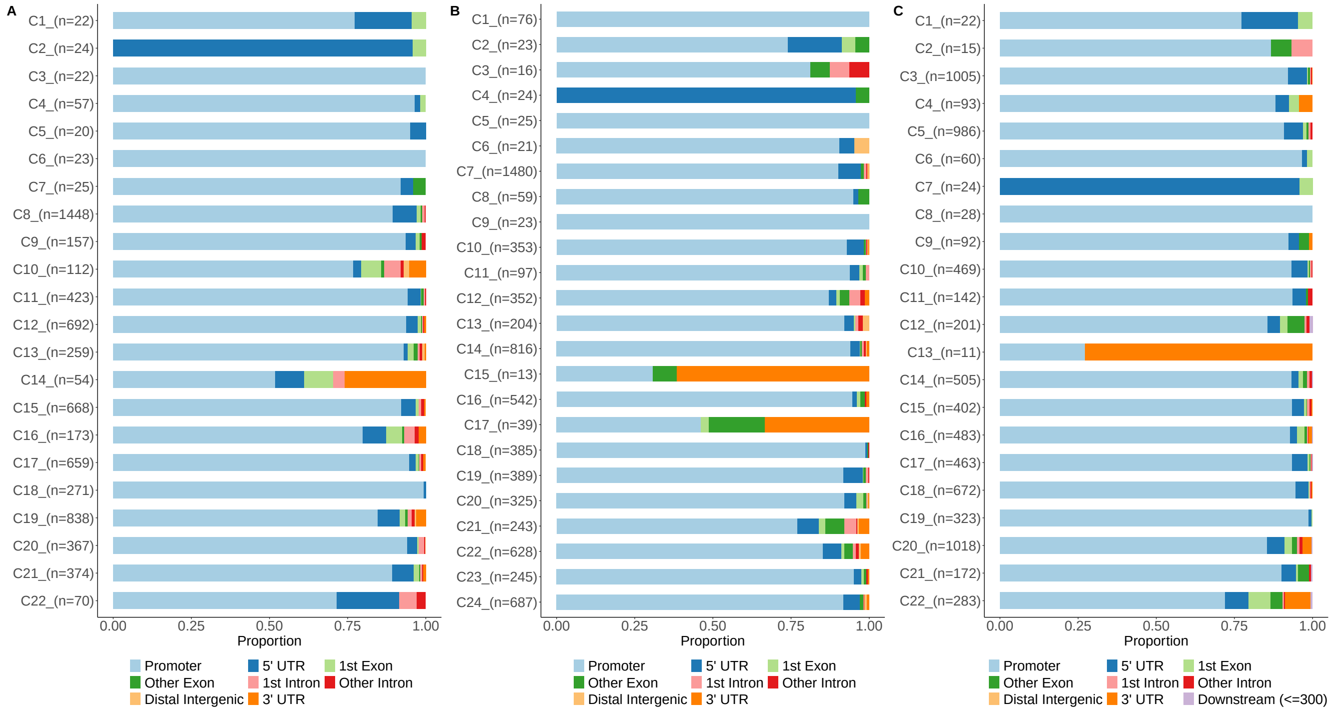

Fig J. Barplots showing proportions of genomic annotations for promoters in all seqArchR clusters in D. melanogaster results for all three stages analysed. (A) 2-4h, (B) 6-8h, and (C) 10-12h AEL

4.4 seqArchR results for D. melanogaster using stability bound 10^{-8}

This section shows the seqArchR results for all stages analysed in D. melanogaster using the bound value 10^{-8}. Based on the observation that some TATA-box architecture promoters were misclassified, we further processed the final collated clusters with lenient bound values to tease out these misclassified architectures. This is detailed in section "Ensuring identification of TATA-box at all stages in D. melanogaster development").

The original result clusters (shown here in Figures Fig K, Fig L, and Fig M) are then updated to accommodate the misclassified TATA architecture clusters. The updated clusters are shown in the main text figures 4, 5, and 6.

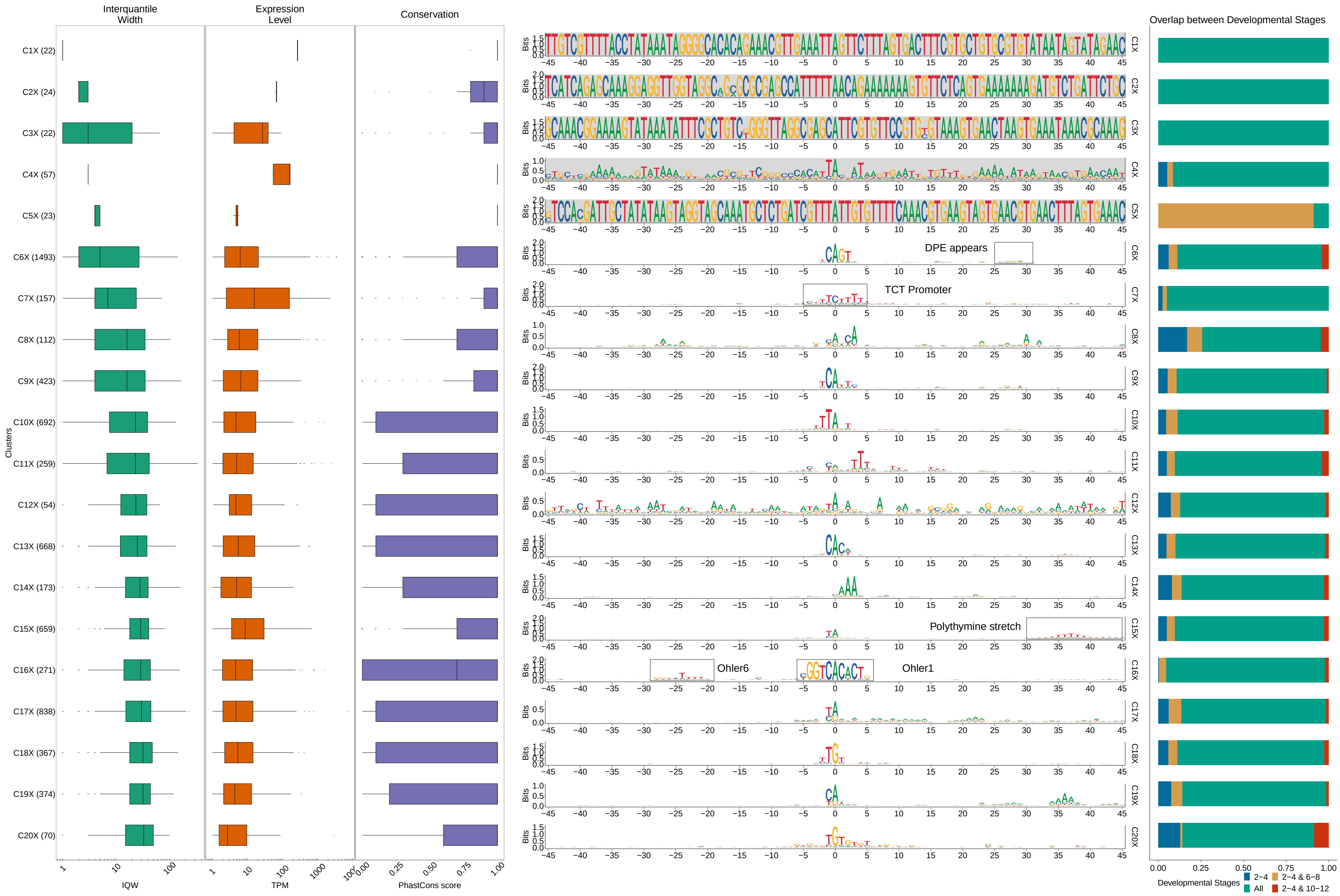

Fig K. Clusters and architectures identified by seqArchR for ***D. melanogaster*, 2-4h AEL**. Sequence clusters arranged by the median interquantile widths (IQW) of CAGE TCs in seqArchR clusters (shortest on top, broadest at the bottom). From left to right: Box and whisker plots of per-cluster IQWs, TPMs, and PhastCons scores followed by per-cluster sequence logos, and stacked barplots showing proportion of TCs unique/shared between transitions. Sequence logos for histone gene clusters are shown with a grey background. 'All' denoting common between all stages. TPM, Tags per million.

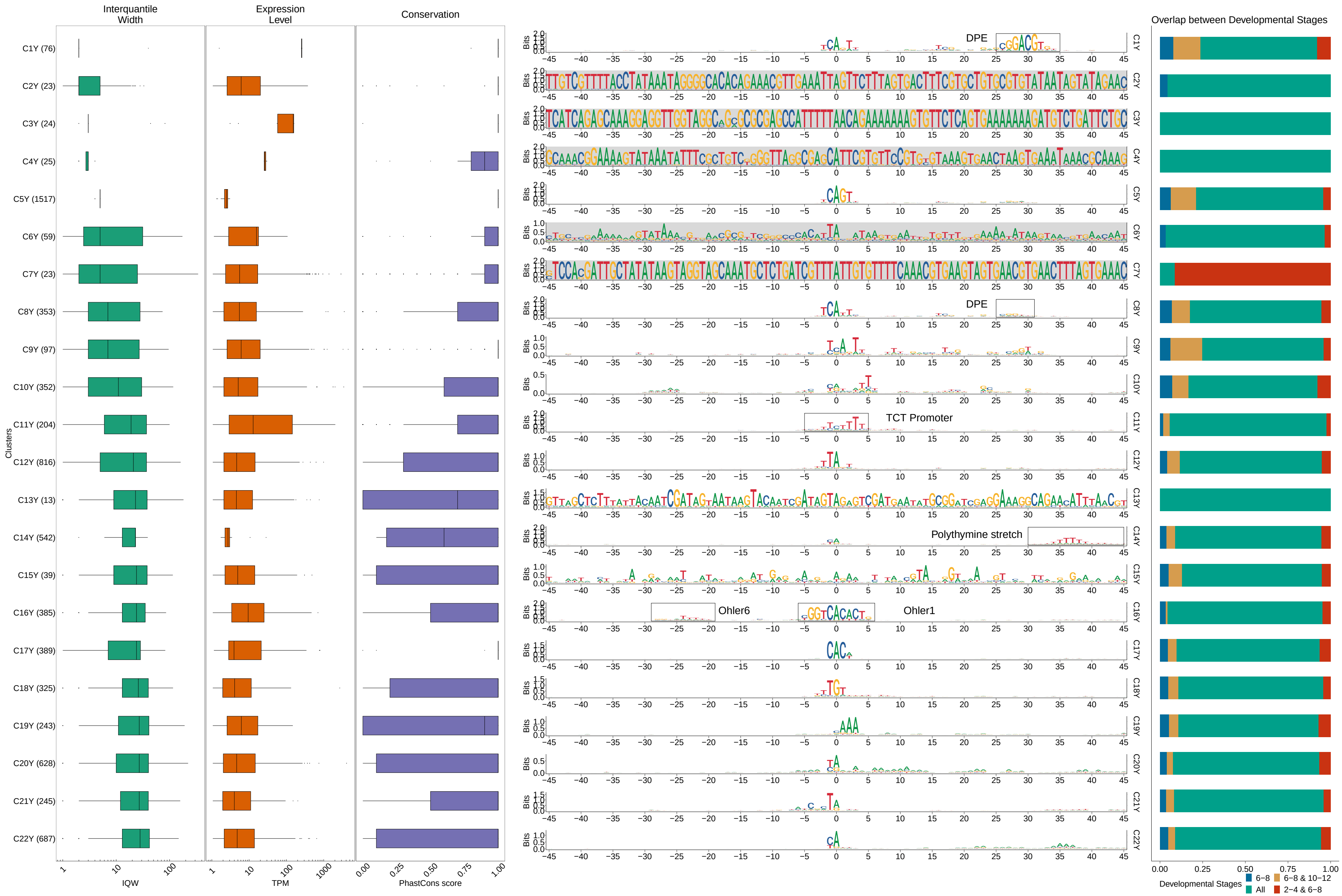

Fig L. Clusters and architectures identified by seqArchR for ***D. melanogaster*, 6-8h AEL**. Sequence clusters arranged by the median interquantile widths (IQW) of CAGE TCs in seqArchR clusters (shortest on top, broadest at the bottom). From left to right: Box and whisker plots of per-cluster IQWs, TPMs, and PhastCons scores followed by per-cluster sequence logos, and stacked barplots showing proportion of TCs unique/shared between transitions. Sequence logos for histone gene clusters are shown with a grey background. 'All' denoting common between all stages. TPM, Tags per million.

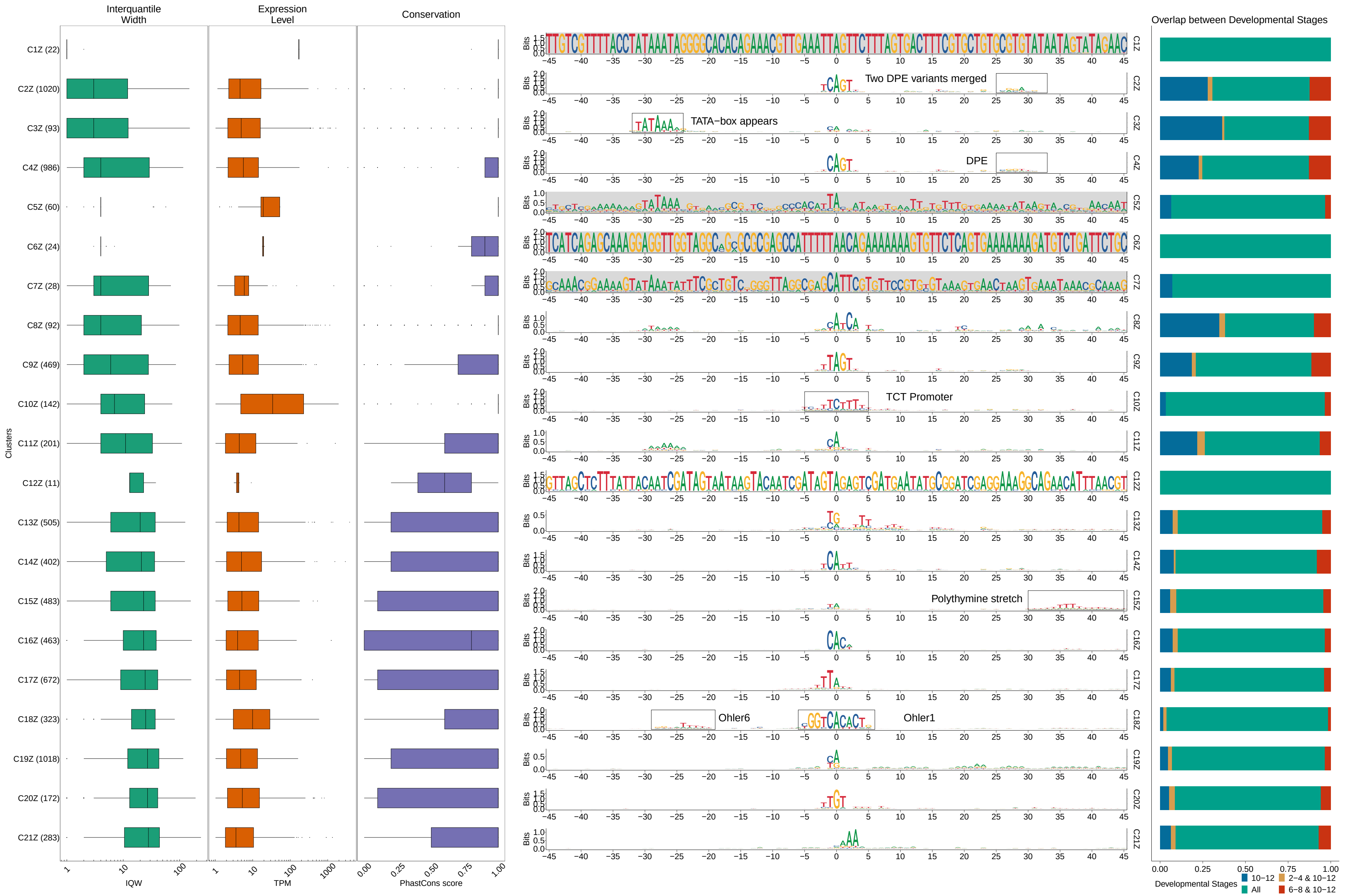

Fig M. Clusters and architectures identified by seqArchR for ***D. melanogaster*, 10-12h AEL**. Sequence clusters arranged by the median interquantile widths (IQW) of CAGE TCs in seqArchR clusters (shortest on top, broadest at the bottom). From left to right: Box and whisker plots of per-cluster IQWs, TPMs, and PhastCons scores followed by per-cluster sequence logos, and stacked barplots showing proportion of TCs unique/shared between transitions. Sequence logos for histone gene clusters are shown with a grey background. ‘All’ denoting common between all stages. TPM, Tags per million.

4.5 Ensuring identification of TATA-box at all stages in D. melanogaster development

For Drosophila melanogaster results, `TATA`-box was not observed in any clusters identified by seqArchR for timepoints before 10-12h AEL using the bound value 10^{-8}. To check whether this was indeed the case, we (a) looked at the motif heatmaps of all clusters showing the presence (color) or absence (white) of the TATA-box consensus sequence in the promoters (see sub-section `TATA` motif occurrence heatmaps); (b) scanned the sequences in those clusters (from all stages) where no visible TATA-box is found (see sub-section Scanning for `TATA`-box using FIMO); (c) processed them with seqArchR using relatively lenient stability bounds (see sub-section Re-processing with seqArchR using lenient stability bound).

4.5.1 `TATA` motif occurrence heatmaps

Figures Fig N, Fig O and Fig P show TATA-box consensus sequence (as pattern) occurrence heatmaps.

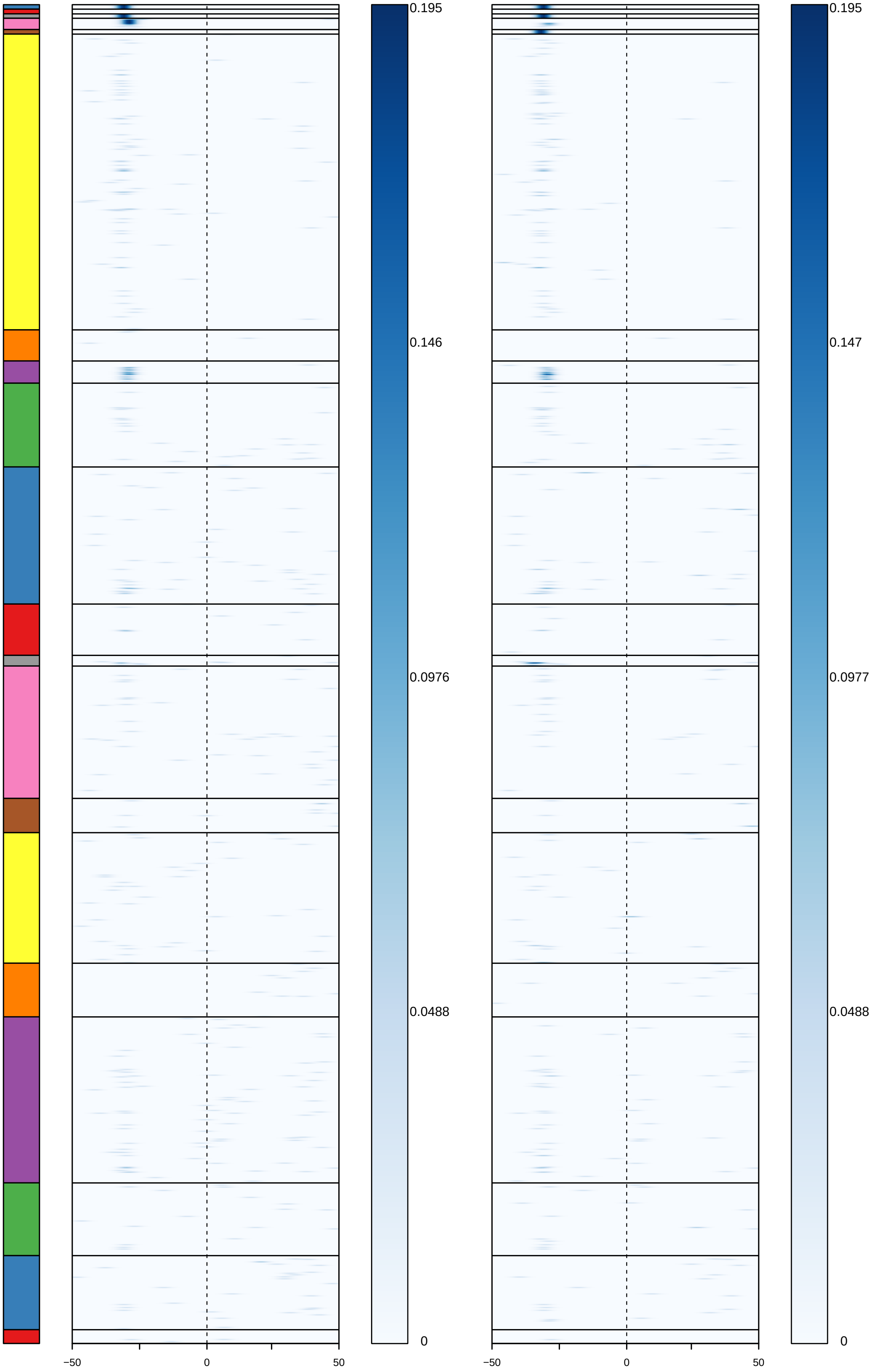

Fig N. Motif heatmaps visualising the presence or absence of the TATA-box consensus sequence in all clusters at 2-4h AEL in D. melanogaster. TATAAA is shown on the left, TATAWAWR is shown on the right.

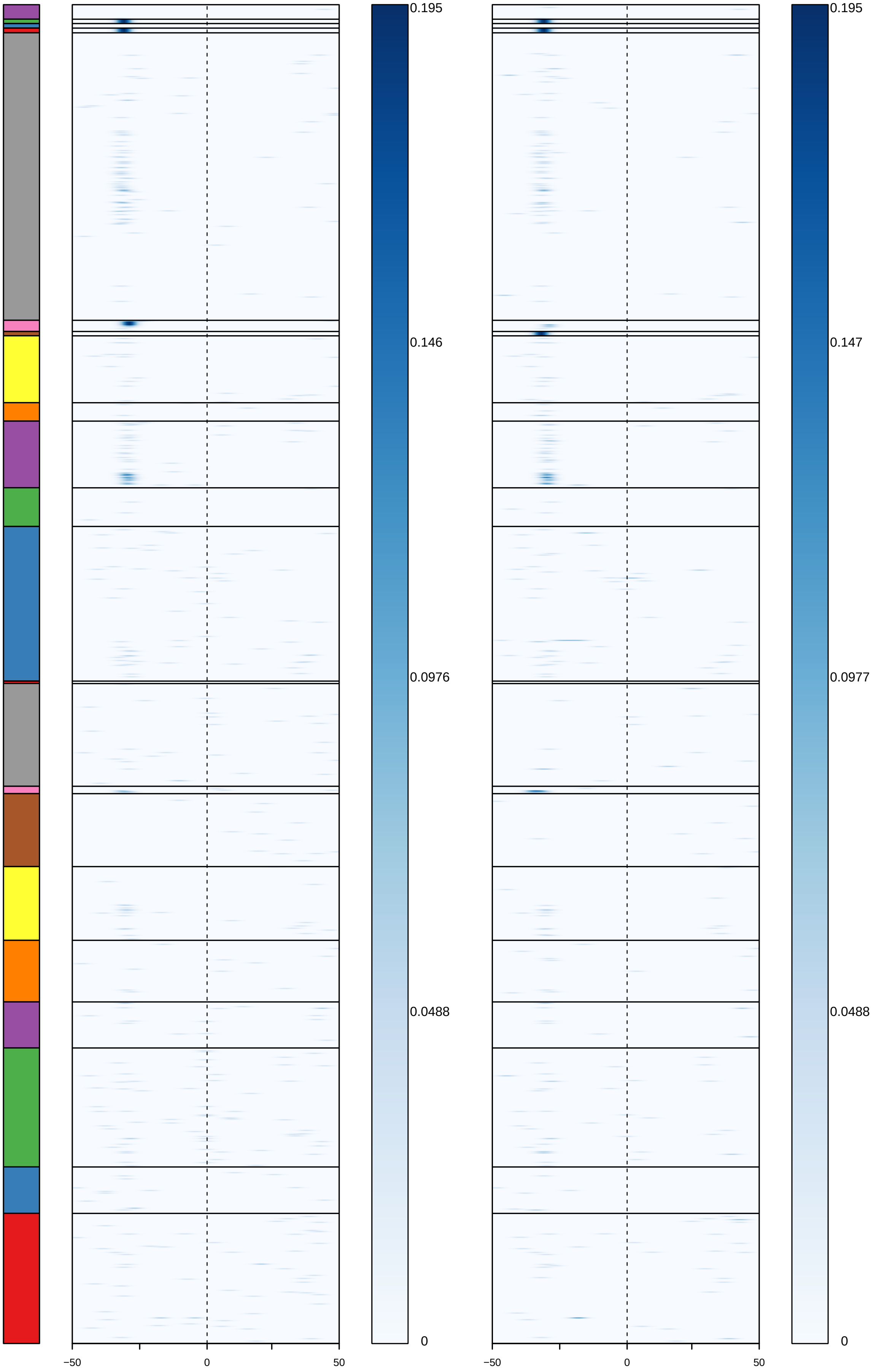

Fig O. Motif heatmaps visualising the presence or absence of the TATA-box consensus sequence in all clusters at 6-8h AEL in D. melanogaster. TATAAA is shown on the left, TATAWAWR is shown on the right.

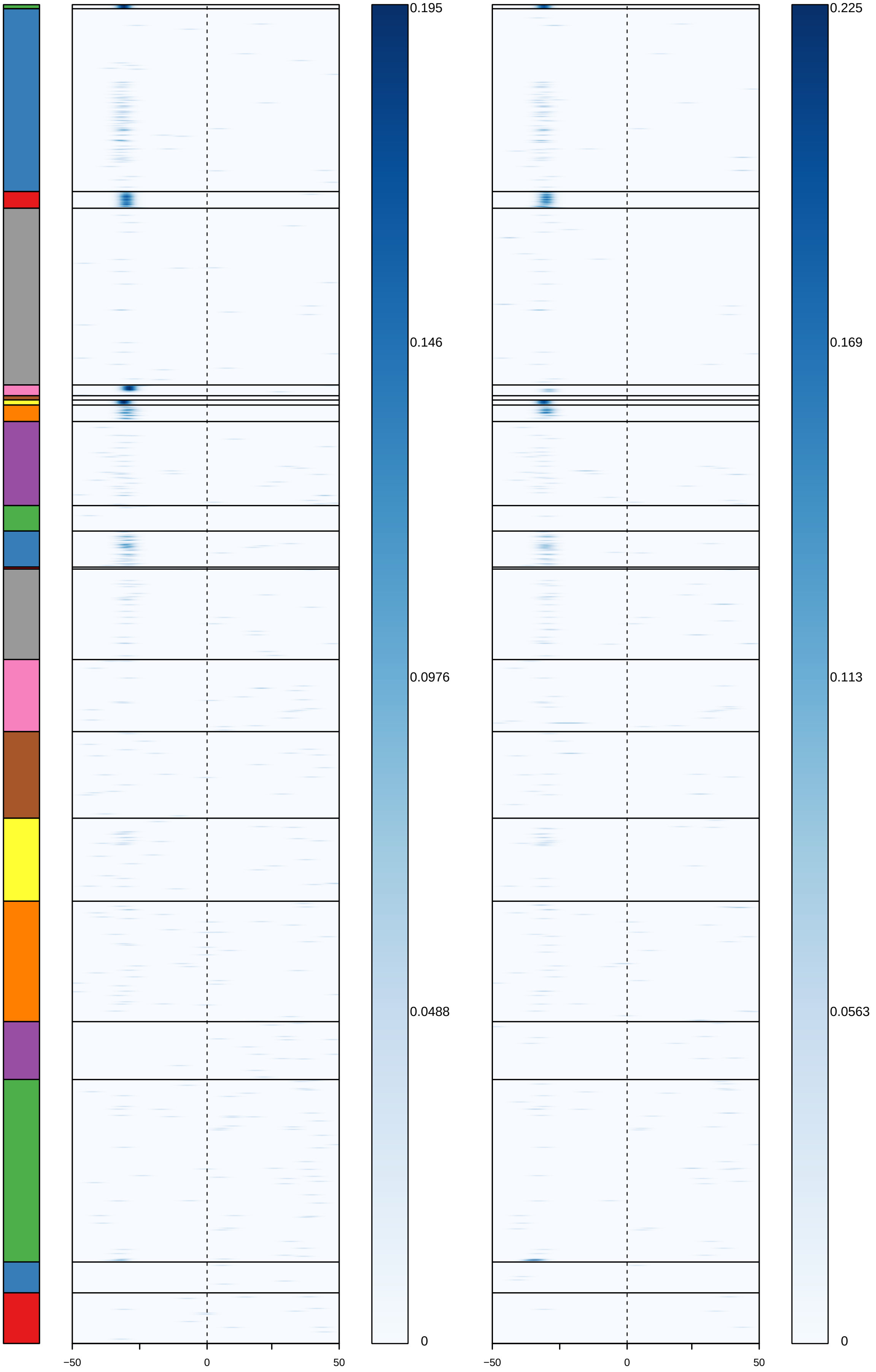

Fig P. Motif heatmaps visualising the presence or absence of the TATA-box consensus sequence in all clusters at 10-12h AEL in D. melanogaster. TATAAA is shown on the left, TATAWAWR is shown on the right.

4.5.2 Scanning for TATA-box using FIMO

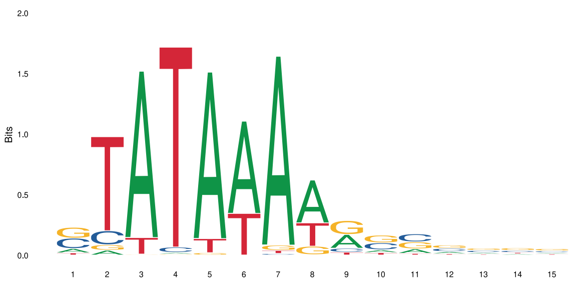

Fig Q. ‘TATA‘-box PWM from JASPAR 2022

Additionally, we used FIMO v4.11.2 ([4]) to scan for the TATA-box PWM (Figure Fig Q; obtained from JASPAR ([5])) in the candidate sequences as follows. For promoter sequences at each stage, those in clusters where seqArchR already identified the TATA-box as part of architectures were separated from those from all other clusters (see Table B below). We call these two sets as SITS (seqArchR-identified TATA sequences) and non-SITS. FIMO was run on both the sets per stage.

Table B. Clusters with TATA-box motifs identified by seqArchR at all three developmental stages in D. melanogaster.

Clusters where seqArchR identified TATA-box (SITS)	Stage
C1X, C3-5X, C8X	2-4h AEL
C2Y, C4Y, C6-7Y, C10Y	6-8h AEL
C1Z, C3Z, C5Z, C7-8Z, C11Z	10-12h AEL

The following figures summarise the FIMO motif scanning results for each stage. As also seen in the motif pattern heatmaps above, we indeed observe some TATA-box motif matches from promoters among non-SITS cluster promoter sequences. Figures Fig R, Fig S, and Fig T show the number of motif matches returned by FIMO separately in each non-SITS promoter cluster. .

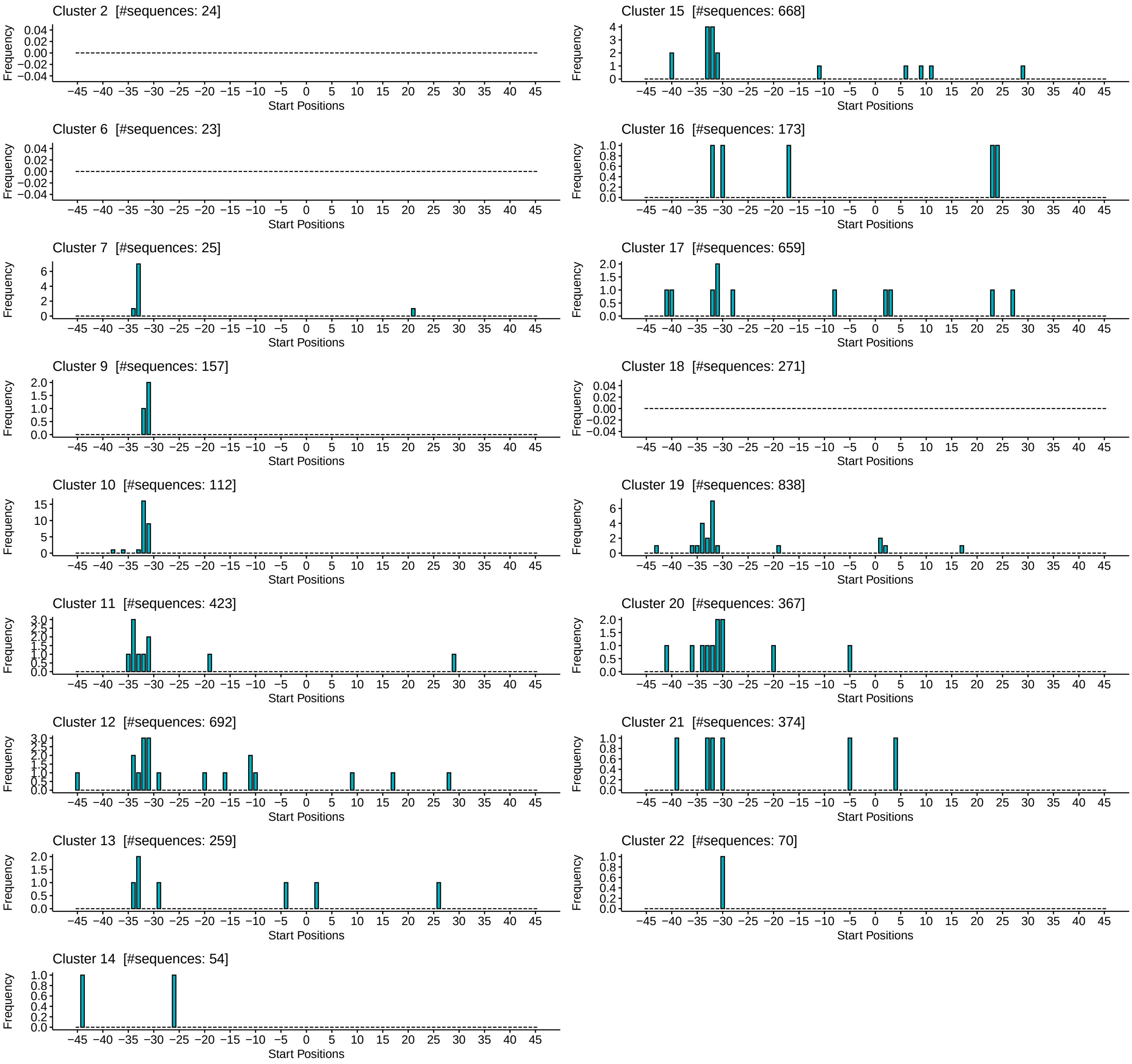

Fig R. Summarising FIMO scan results for 2-4h AEL. Frequency of motif matches (motif starts) at various positions in the individual non-SITS clusters obtained from FIMO scanning results are shown for 2-4h AEL stage

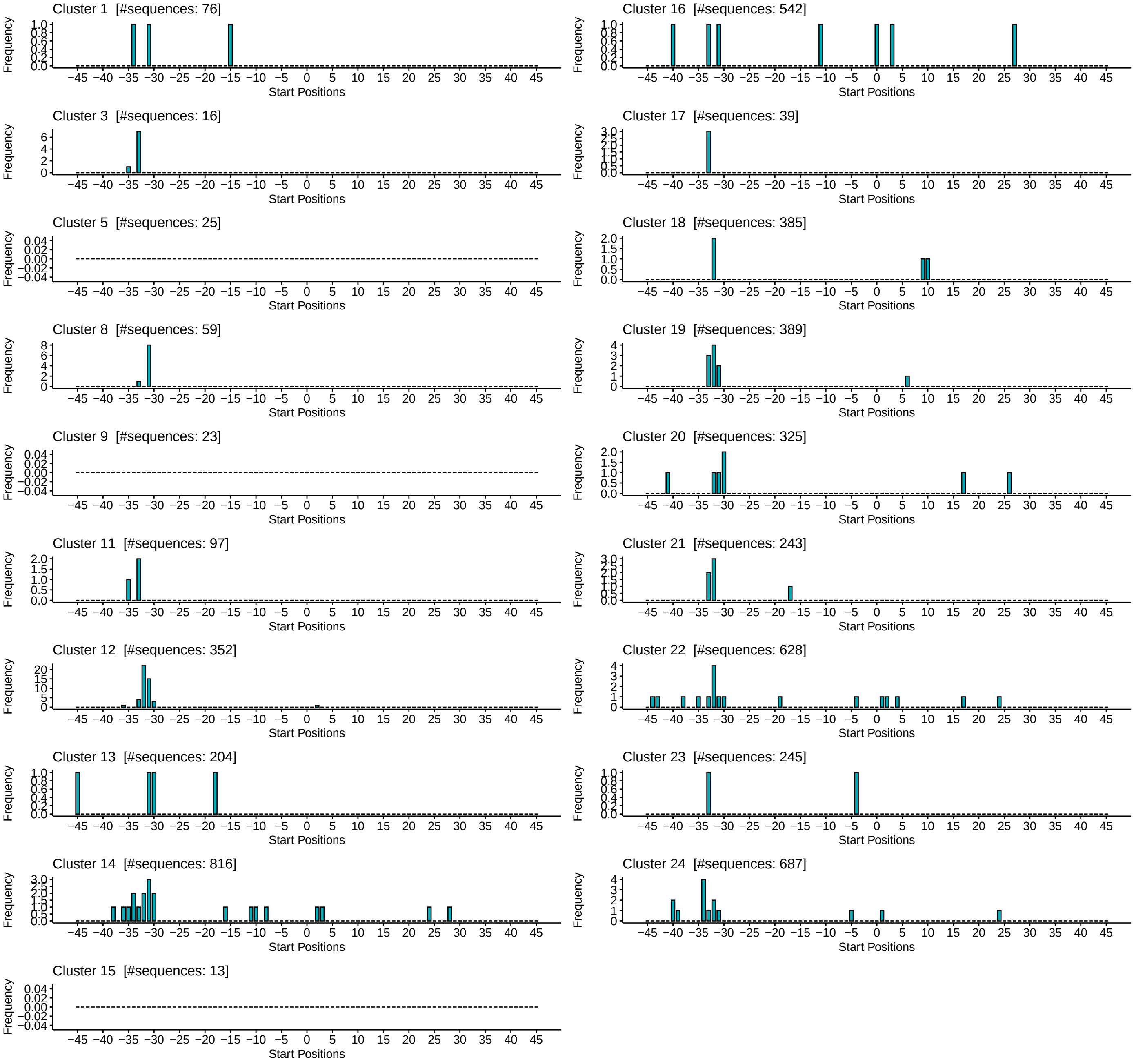

Fig S. Summarising FIMO scan results for 6-8h AEL. Frequency of motif matches (motif starts) at various positions in the individual non-SITS clusters obtained from FIMO scanning results are shown for 6-8h AEL stage

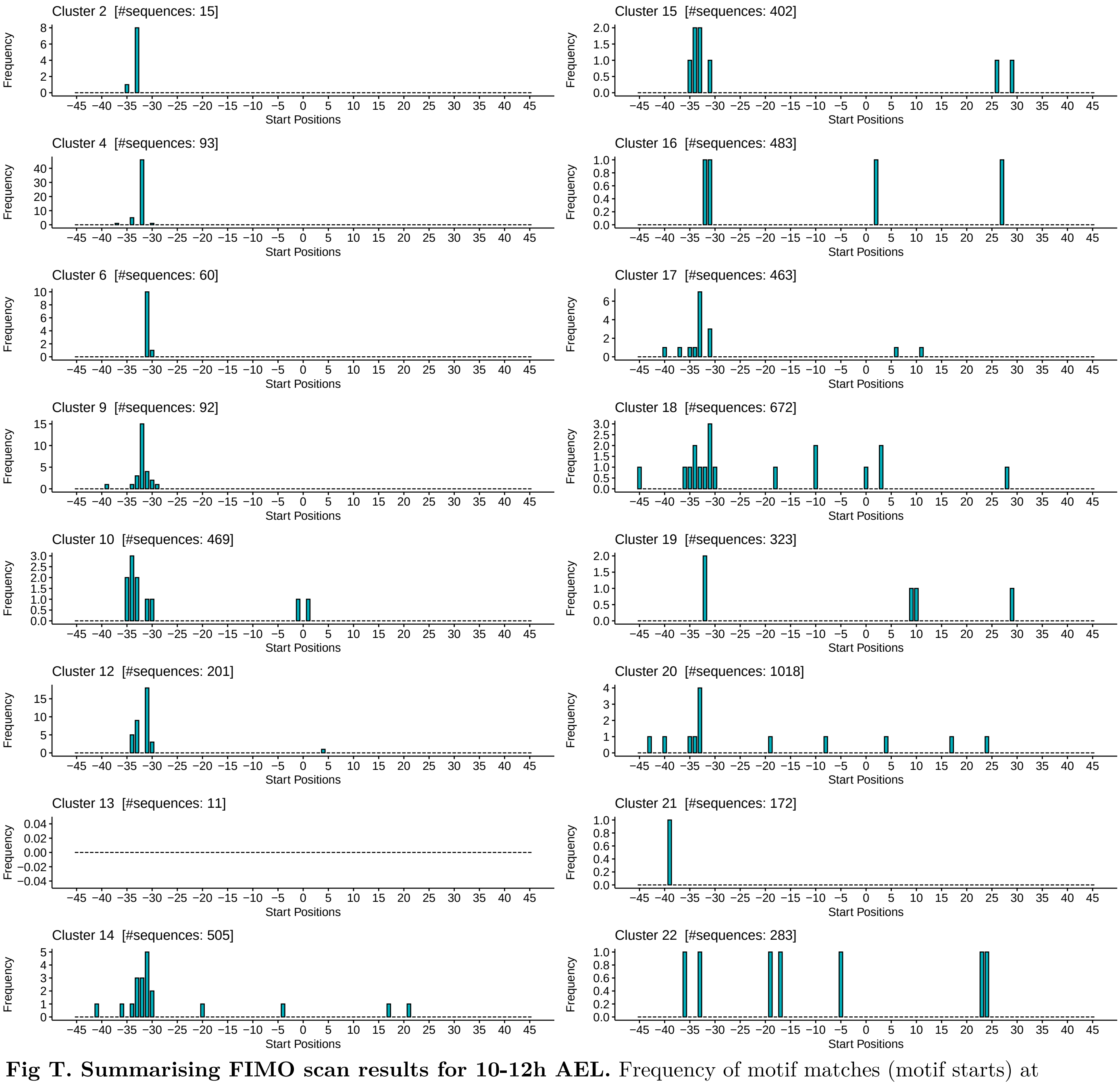

Fig T. Summarising FIMO scan results for 10-12h AEL. Frequency of motif matches (motif starts) at various positions in the individual non-SITS clusters obtained from FIMO scanning results are shown for 10-12h AEL stage

Interestingly, majority of the FIMO matches in stages 2-4h and 6-8h AEL stem from clusters with a strong DPE signal (C6X and C5Y). In fact, even at 10-12h stage, the cluster C2Z, which has two DPE variants, has the majority of the matches found by FIMO. Almost all of these matches occur at start positions $-34/-33$ from the TSS, implying that the first `T`, which is at the second position in the motif, occurs at $-33/-32$ relative to the dominant TSS. Therefore, (a) we consider the `TATA`-box promoters in clusters C6X, C5Y and C2Z as mishits which should have been identified by seqArchR; and (b) as the number of FIMO-found matches in other clusters are significantly lower, we consider them as misclassifications by seqArchR. We next focus on checking if seqArchR can identify the mishits in set (a).

In general, there are two possible reasons for seqArchR to miss this signal. First, they are not very well aligned, and, second, the signal is not strong enough to be identified either by itself or is diminished by the presence of some other relatively stronger signal, like the DPE in the clusters discussed above. This could lead to the `TATA`-box being unidentified and sequences, misclassified. In such scenarios, when the signal is not strong enough, making the stability bound more lenient (see *model selection criterion*, Methods section), can sometimes help.

4.5.3 Re-processing with seqArchR using lenient stability bound

We processed all non-SITS clusters from the final clusters reported for D. melanogaster (all three stages) individually with seqArchR using progressively lenient bound values, $10^{-7,-6,-5}$ compared to 10^{-8} used for the reported result clusters in D. melanogaster. Indeed, the missed `TATA`-box promoters were found by seqArchR for all three stages, specifically in the following three clusters – C6X, C5Y, and C2Z (cluster IDs corresponding to figures Fig F), Fig G, and Fig H. For both C6X and C5Y, seqArchR identified `TATA` sequences at bound value 10^{-6}, and C2Z at bound value 10^{-7}. All further clusters/architectures identified within these clusters are shown in Supplementary figures Fig U, Fig V, and Fig W.

This suggests that, this was indeed the case of the TATA signal, diminished due to the stronger DPE signal, not being identified at the given bound value 10^{-8}.

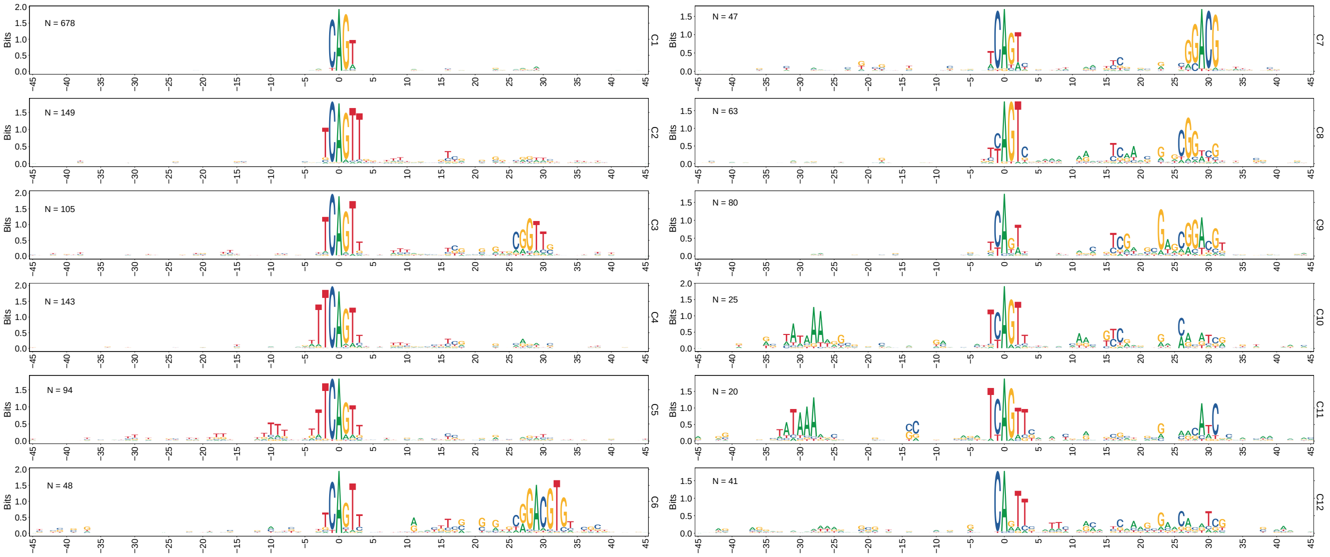

Fig U. Sequence logos of further architectures/clusters identified within cluster C6X at 2-4h stage in D. melanogaster with a lenient bound value

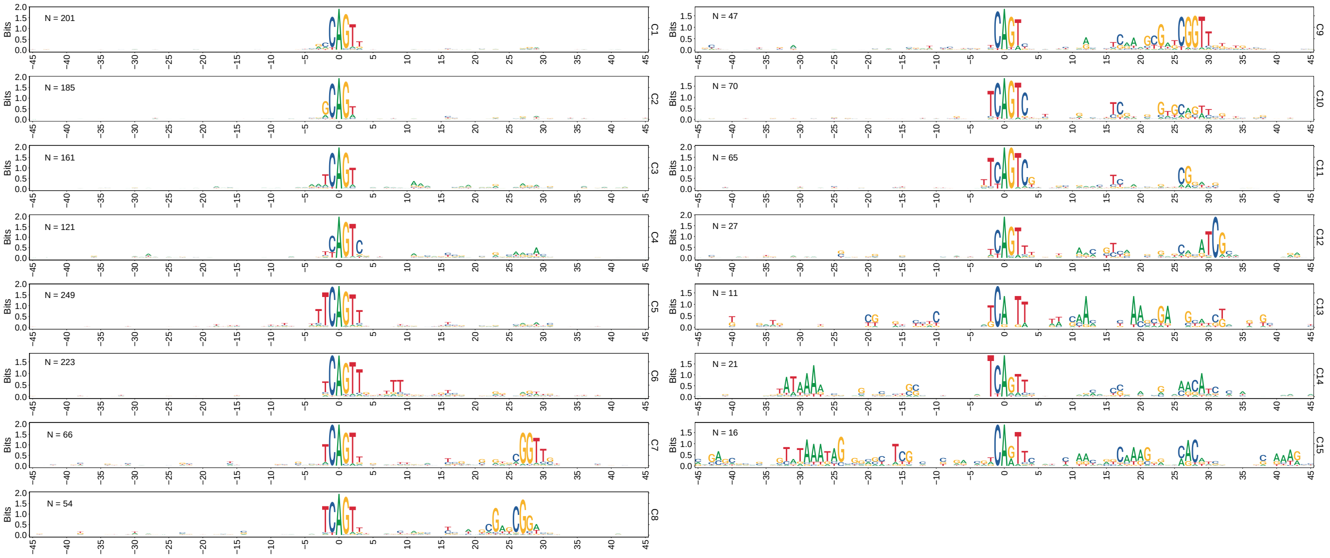

Fig V. Sequence logos of further architectures/clusters identified within cluster C5Y at 6-8h stage in D. melanogaster with a lenient bound value

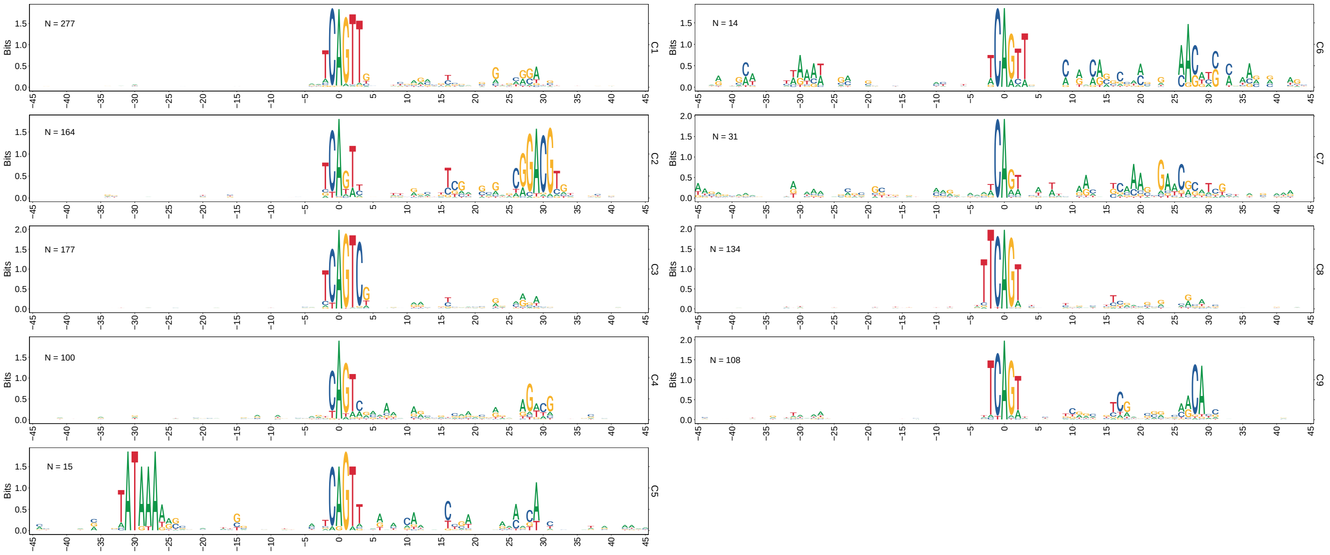

Fig W. Sequence logos of further architectures/clusters identified within cluster C2Z at 10-12h stage in D. melanogaster with a lenient bound value

4.5.4 Summary

In summary, seqArchR could indeed identify most of the misclassified TATA-box promoters hiding in clusters with the DPE motif. Note that these TATA-boxes appear at -32 or -33 bp upstream of the dominant TSS, instead of -31 bp as seen in the cluster C3Z (10-12h) reported in the main text, Figure 6.

As discussed in the main article (see Discussion section), in the future, it may be beneficial to adaptively change the bound value for the different iterations or clusters.

4.6 GO terms enriched for different clusters in Drosophila melanogaster development

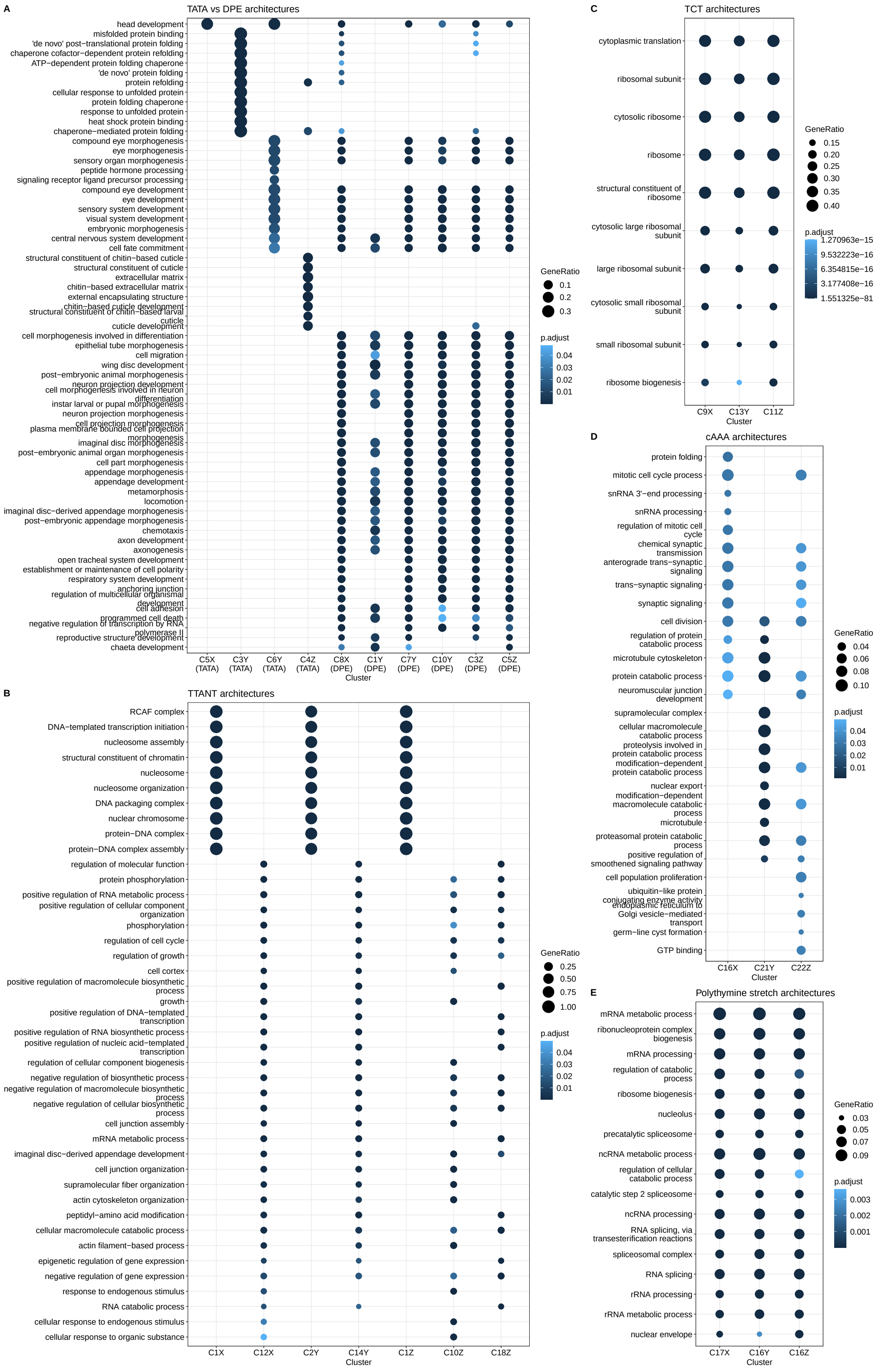

Fig X. Visualisation of top-10 GO terms enriched for various clusters at different developmental stages of Drosophila melanogaster.

5 Supplementary figures of results on Zebrafish core promoters from [6]

5.1 Visualisation showing curation of seqArchR result clusters for D.rerio

In Figures Fig Y, Fig Z, and Fig AA, we show the curation of raw clusters (of promoter sequences) from seqArchR result for the different stages processed for Danio rerio. Note that, here, the clusters are shown ordered by the hierarchical clustering dendrogram (sequence logos on the left). For the sequence logos on the right, the collated clusters are ordered by their IQW. This is the same ordering as in the figures in the main text, including the cluster names.

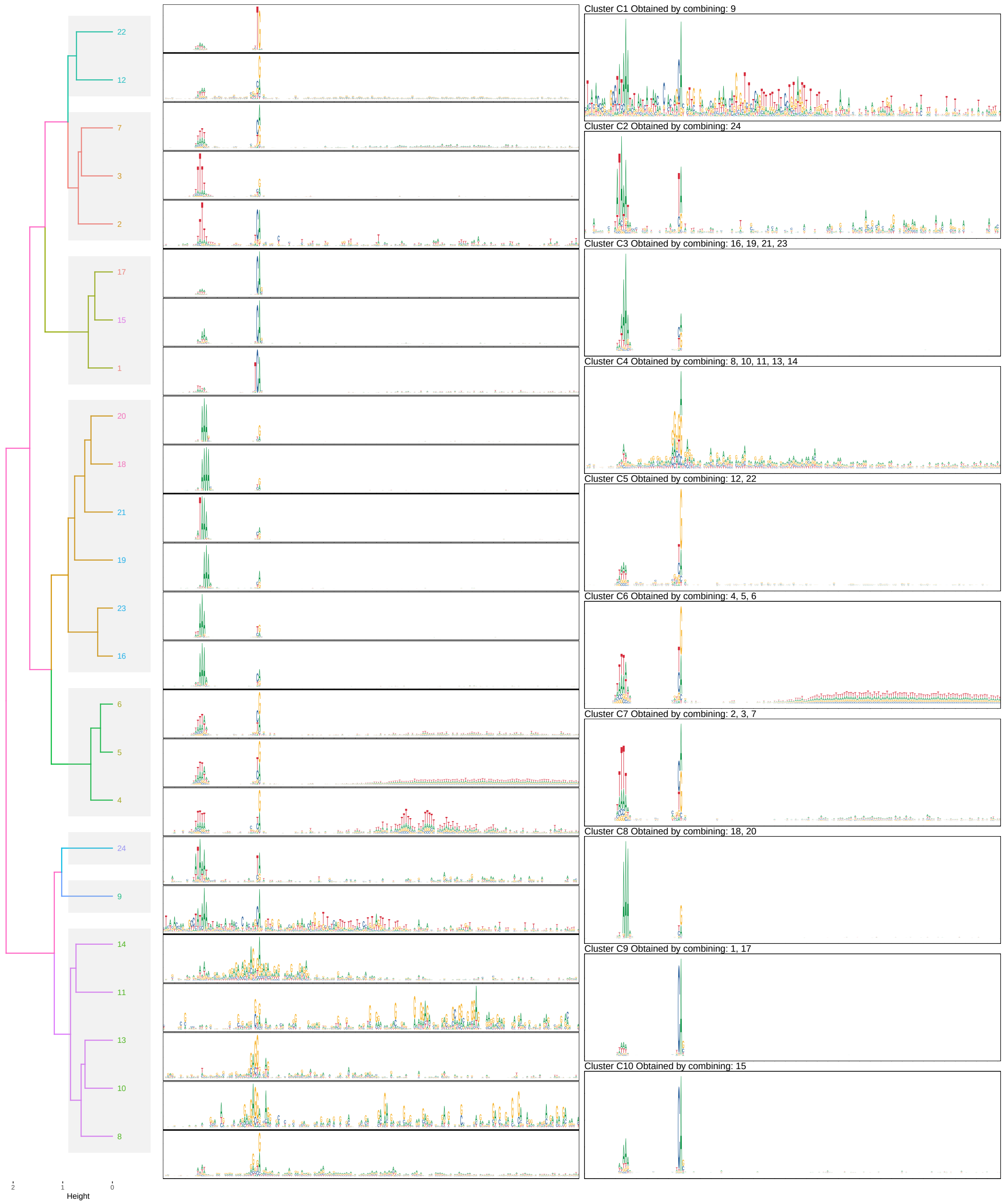

Fig Y. Visualisation of the collation and curation of clusters from seqArchR raw result for 64 cells stage, D.rerio

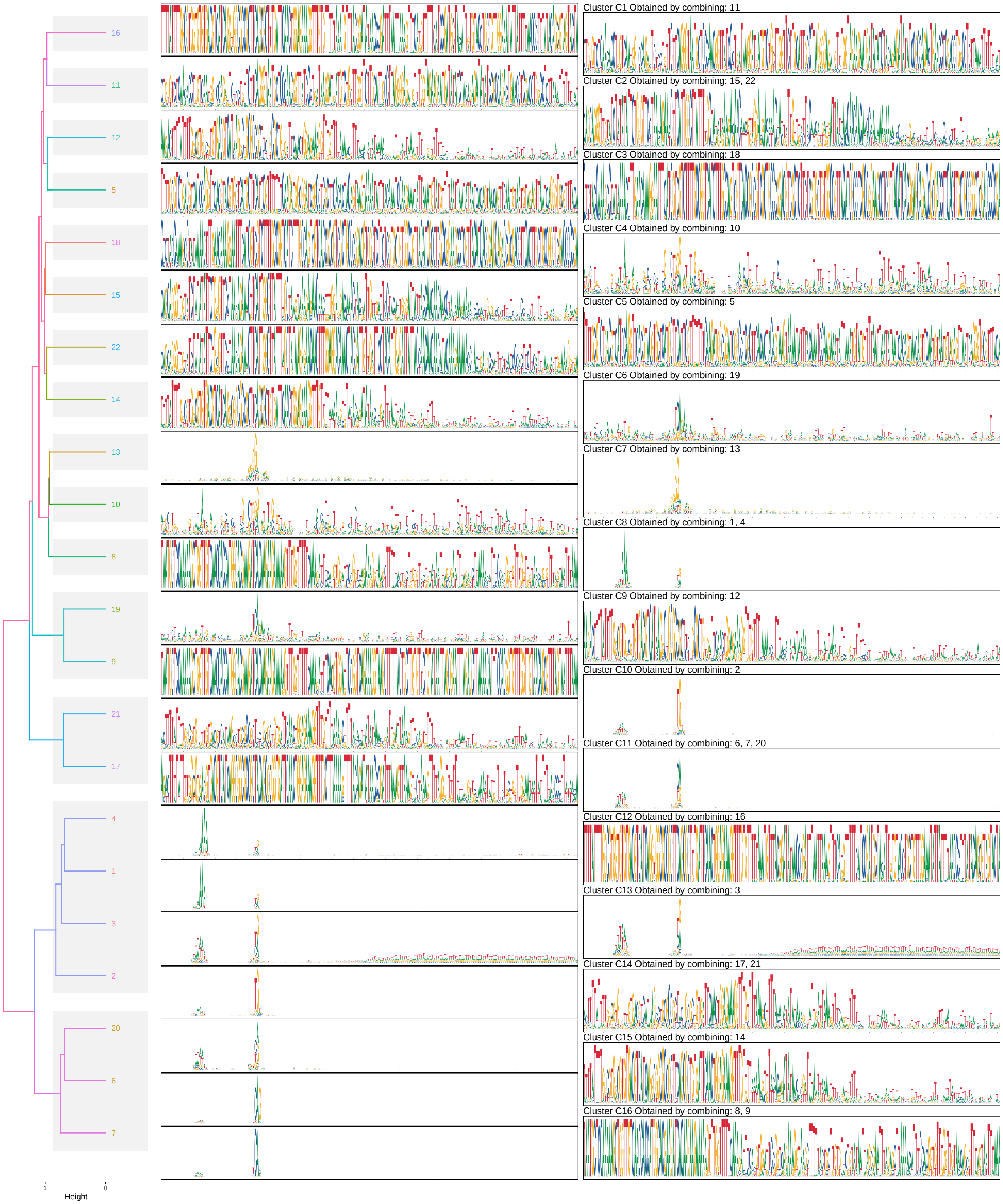

Fig Z. Visualisation of the collation and curation of clusters from seqArchR raw result for 30% Epiboly/Dome stage, D.rerio

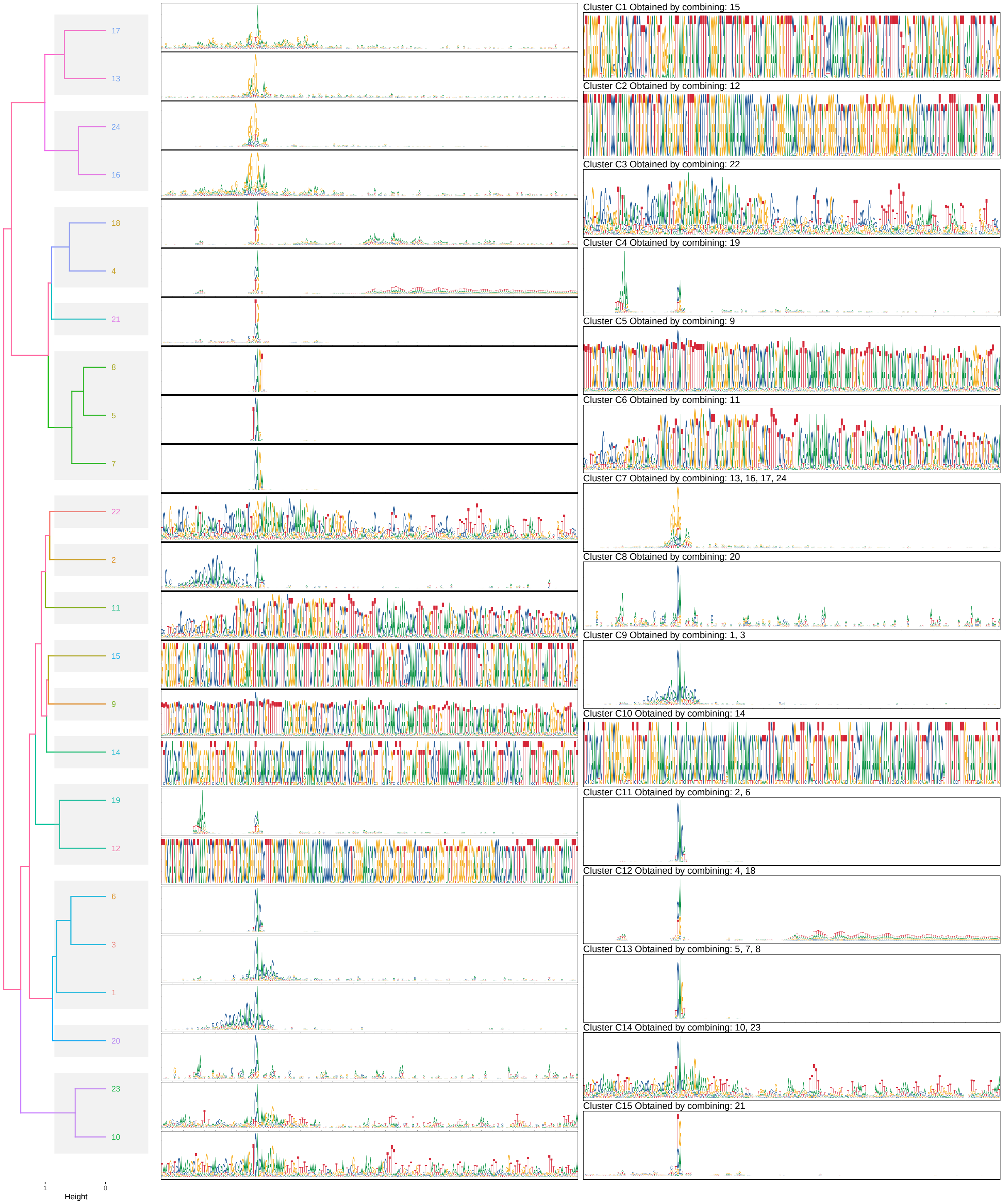

Fig AA. Visualisation of the collation and curation of clusters from seqArchR raw result for Prim-6, D.rerio

5.2 Motif heatmaps for Zebrafish development stages

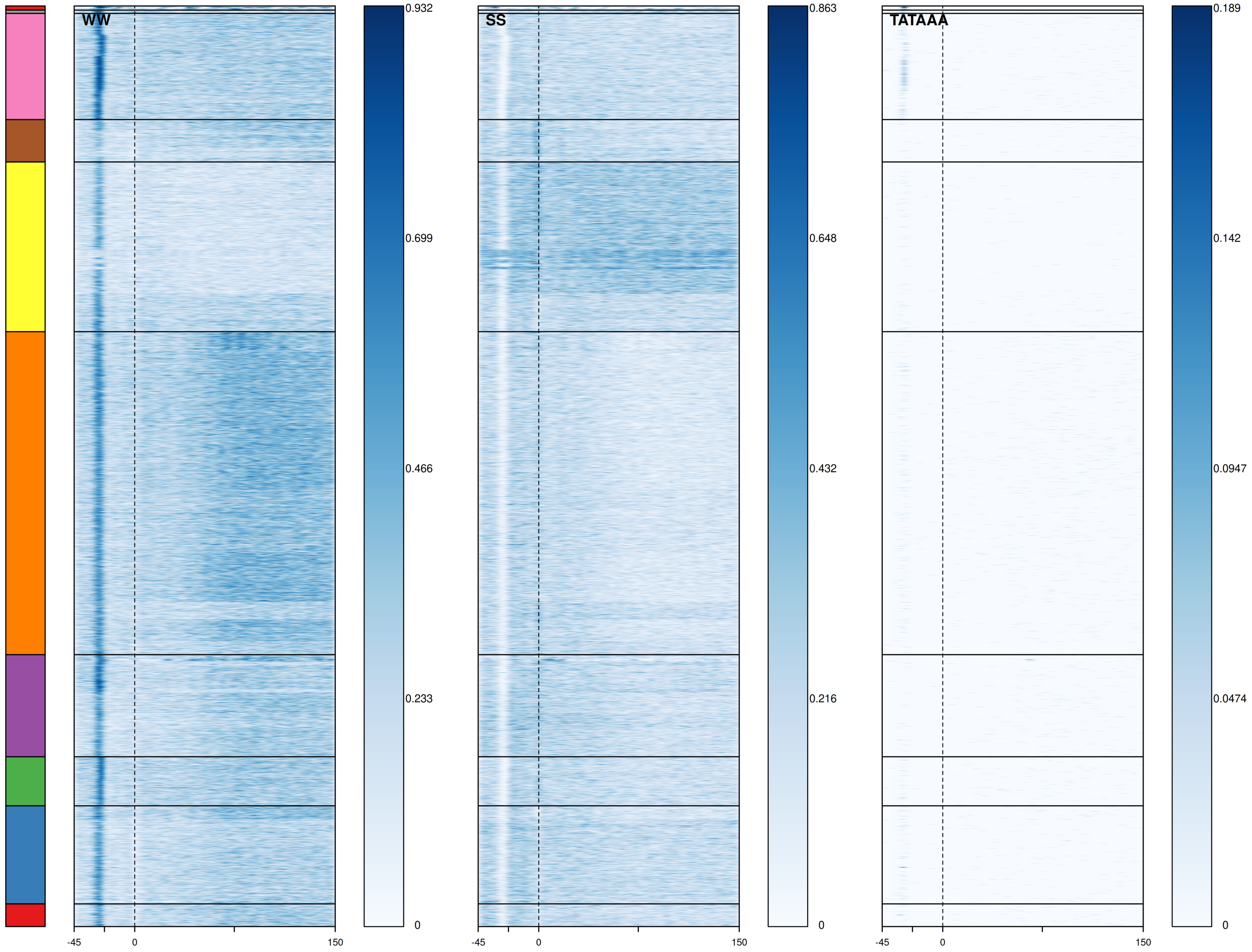

Fig AB. Heatmaps showing occurrences of motif words among seqArchR clusters for 64 cells stage, D.rerio

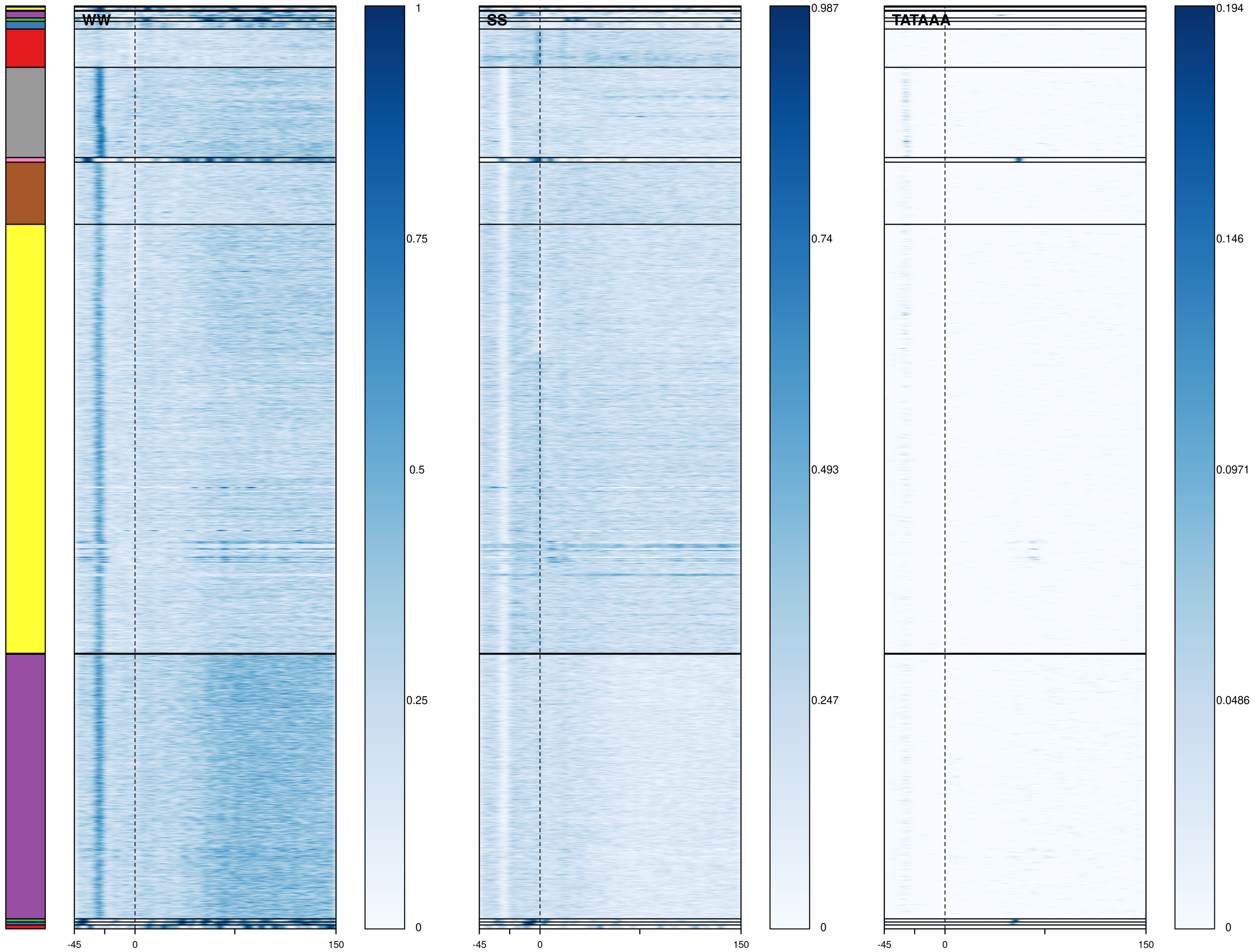

Fig AC. Heatmaps showing occurrences of motif words among seqArchR clusters for 30% Epiboly/Dome stage, D.rerio

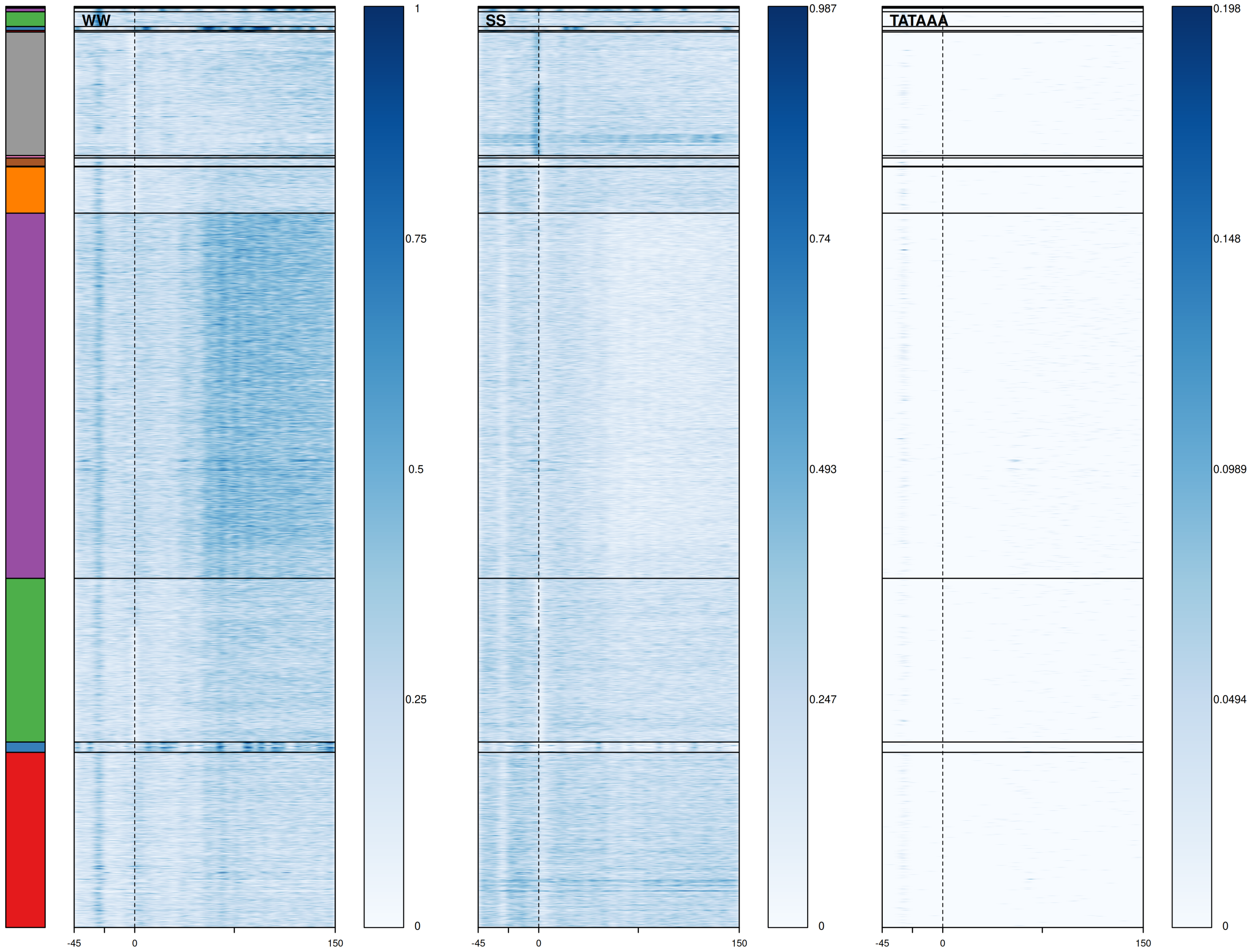

Fig AD. Heatmaps showing occurrences of motif words among seqArchR clusters for Prim-6 stage, D.rerio

5.3 Chromosomal locations of promoters/CTSSs in seqArchR clusters for Zebrafish development stages

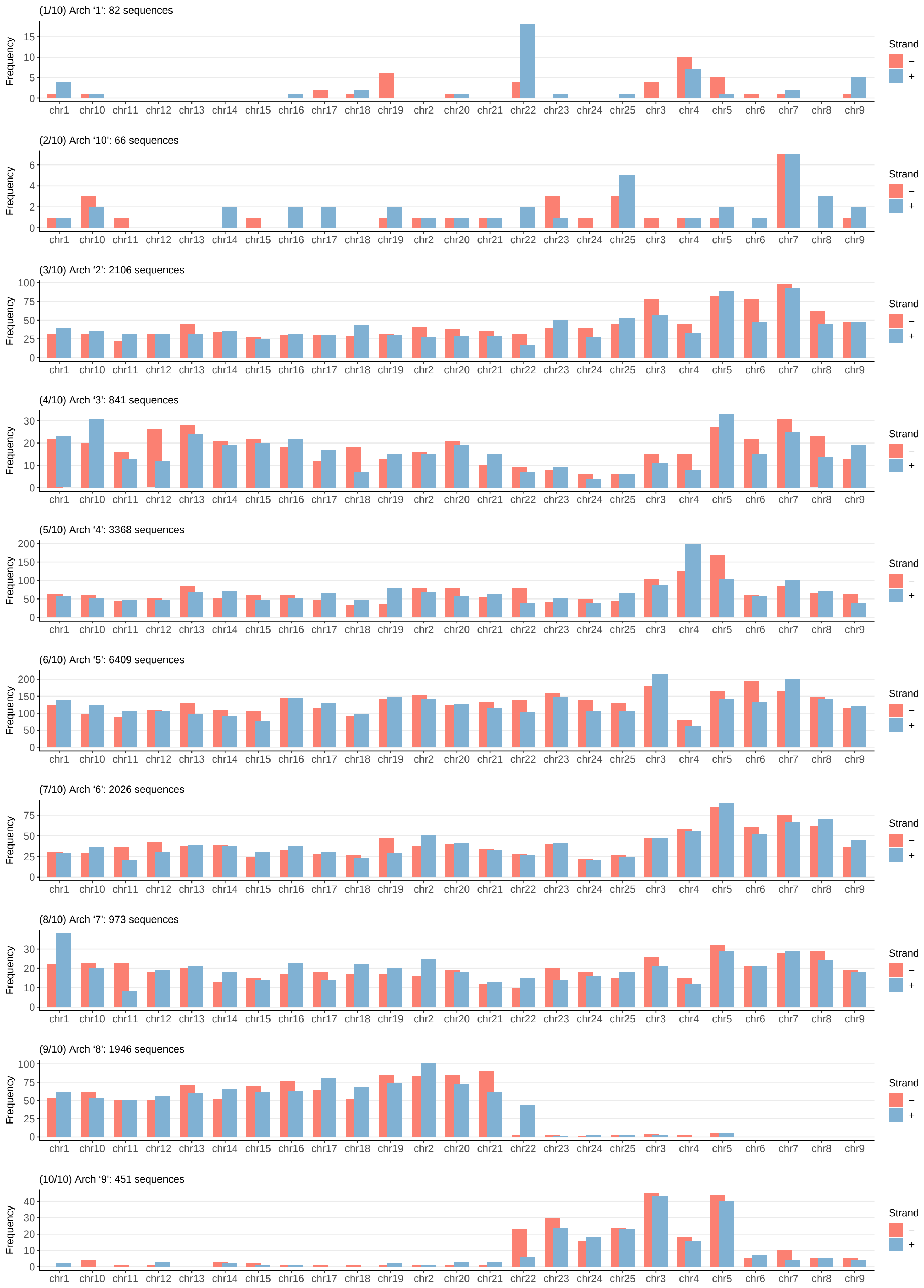

Fig AE. Chromosomal locations of promoters/CTSSs in seqArchR clusters for 64 cells stage, D.rerio

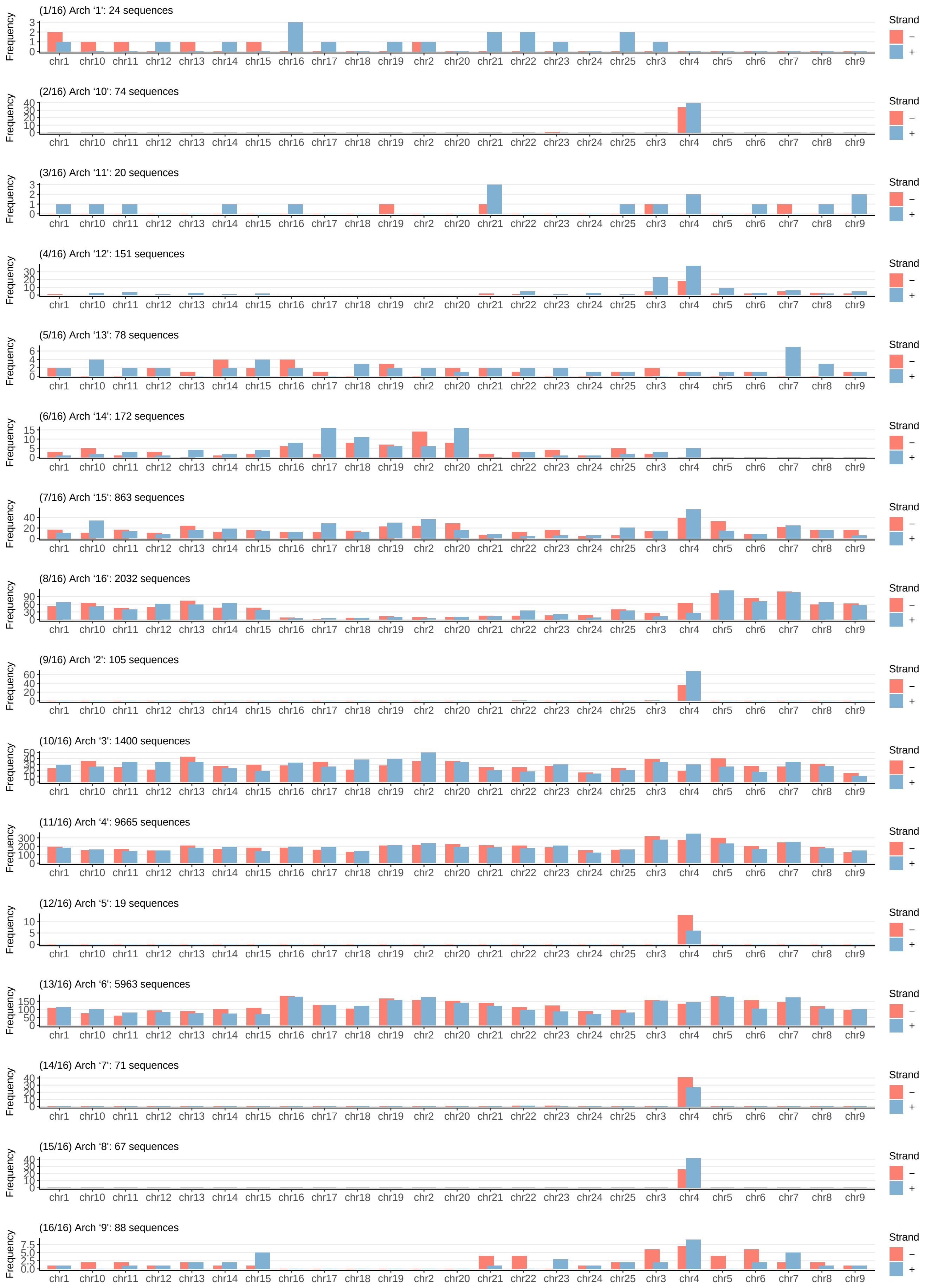

Fig AF. Chromosomal locations of promoters/CTSSs in seqArchR clusters for 30% Epiboly/Dome stage, D.rerio

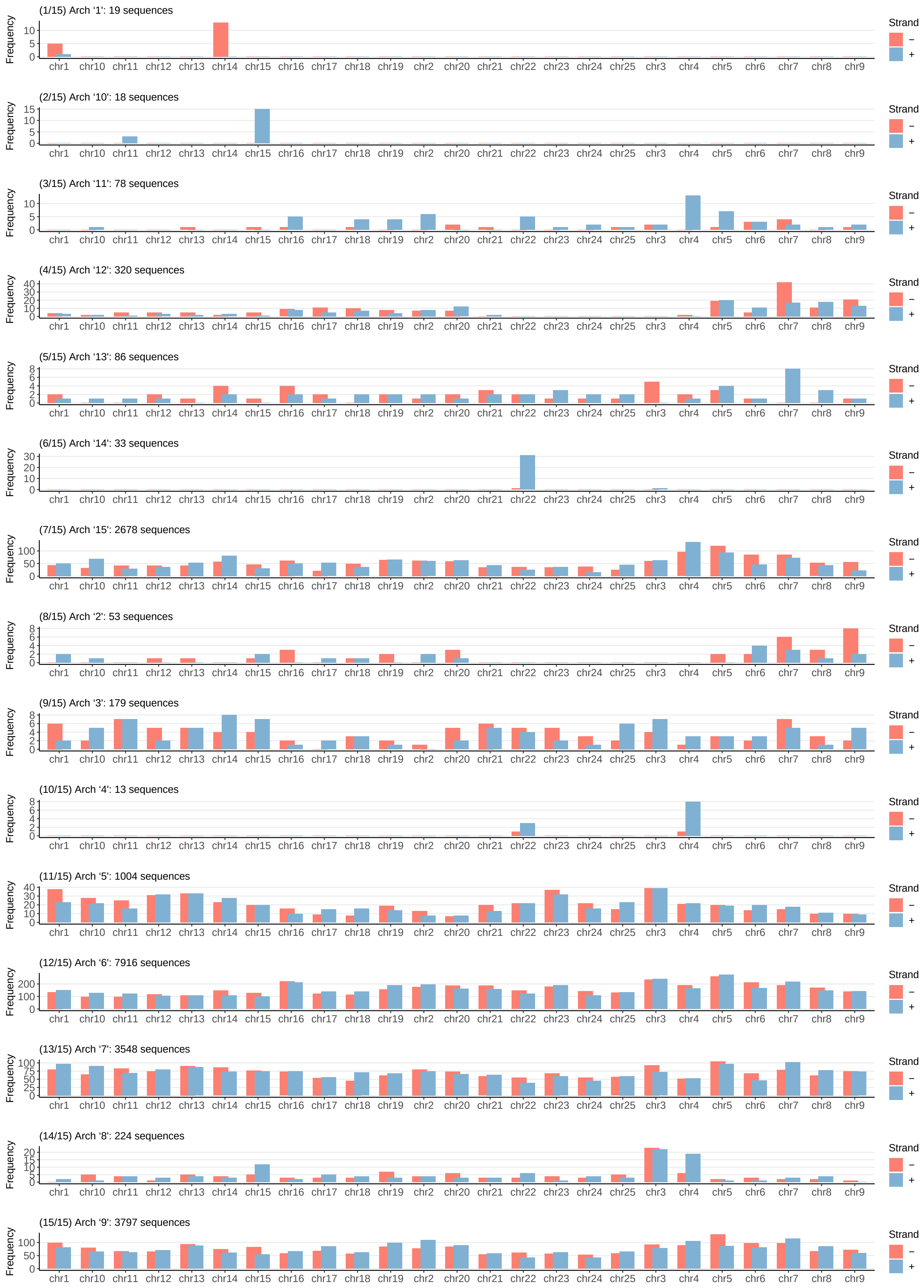

Fig AG. Chromosomal locations of promoters/CTSSs in seqArchR clusters for Prim-6 stage, D.rerio

5.4 Overlaps between promoter sequences at all stages in D.rerio

Similar to the rightmost panel in figures showing Drosophila results, we visualise the overlaps between tag clusters at every developmental stage in zebrafish in Figure Fig AH.

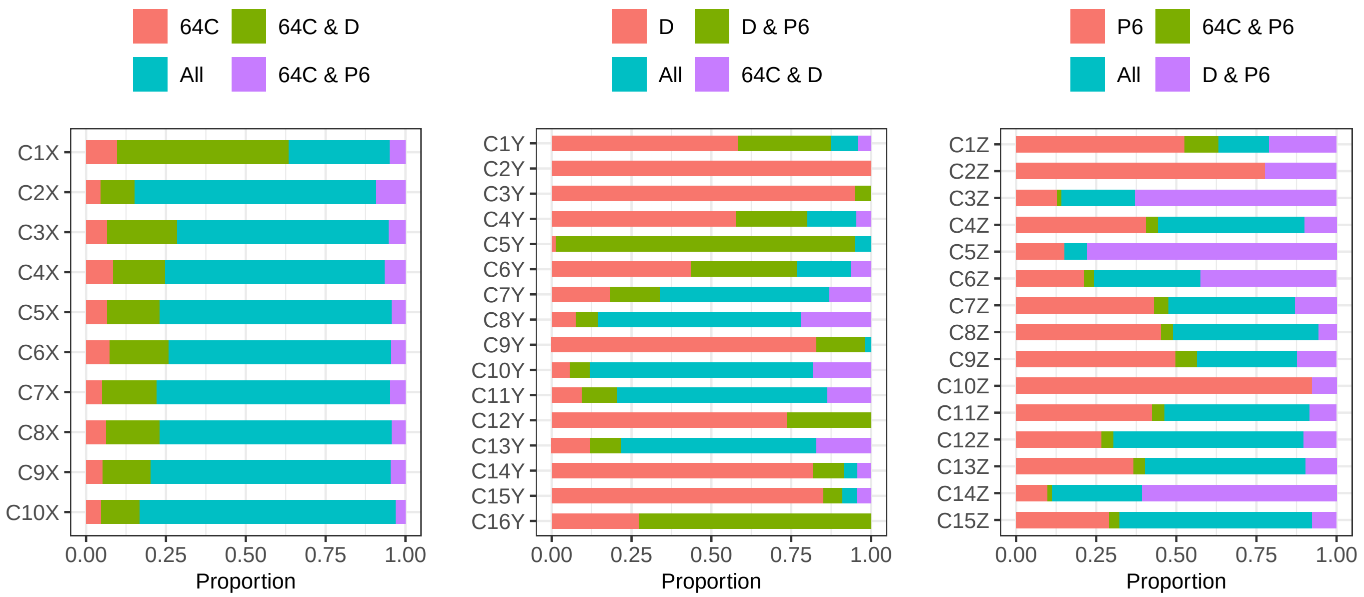

Fig AH. Visualisation of the overlaps between promoter sequences per developmental stage for D.rerio. Stages are abbreviated as follows. 64C: 64 cells stage; D: 30% Epiboly/Dome stage; P6: Prim-6 stage.

5.5 Top-10 GO term enrichments for clusters

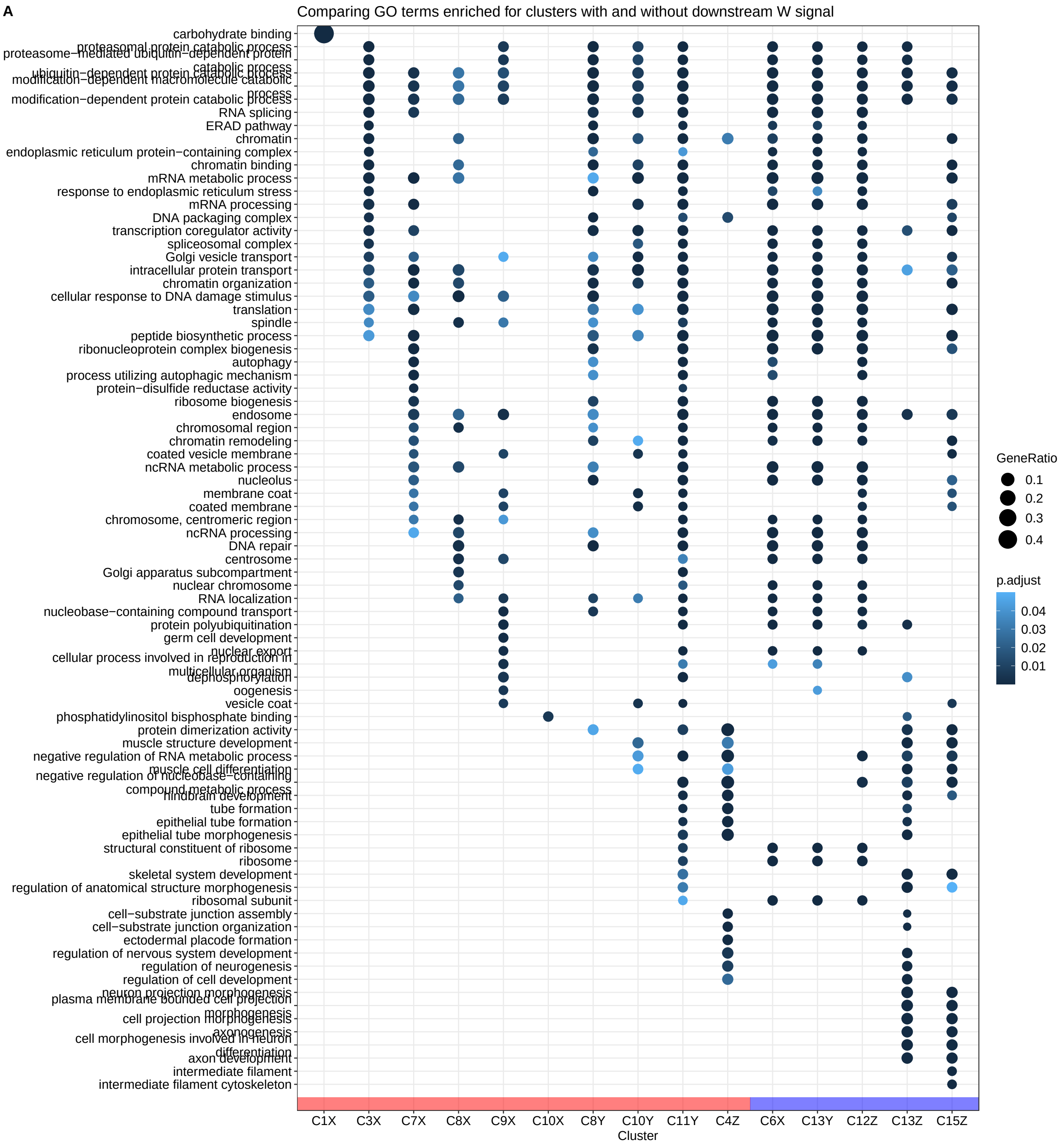

Fig AI. Top-10 enriched GO terms for selected clusters across ZF developmental stages.

6 Supplementary figures of results on human cell lines core promoters from ENCODE

We processed promoter sequences obtained using ENCODE CAGE data in human cell lines and tissues. Figure Fig AJ shows the library sizes of various cell lines (including replicates) obtained from ENCODE and used upon merging together. The experiments were performed with different sizes of flanks around the TSS. Specifically, upstream flank of 50 bp was combined with a short, 5 bp flank and a longer, 150 bp, flank downstream. The rationale for this is explained in the main text, the Results sub-section on Homo sapiens.

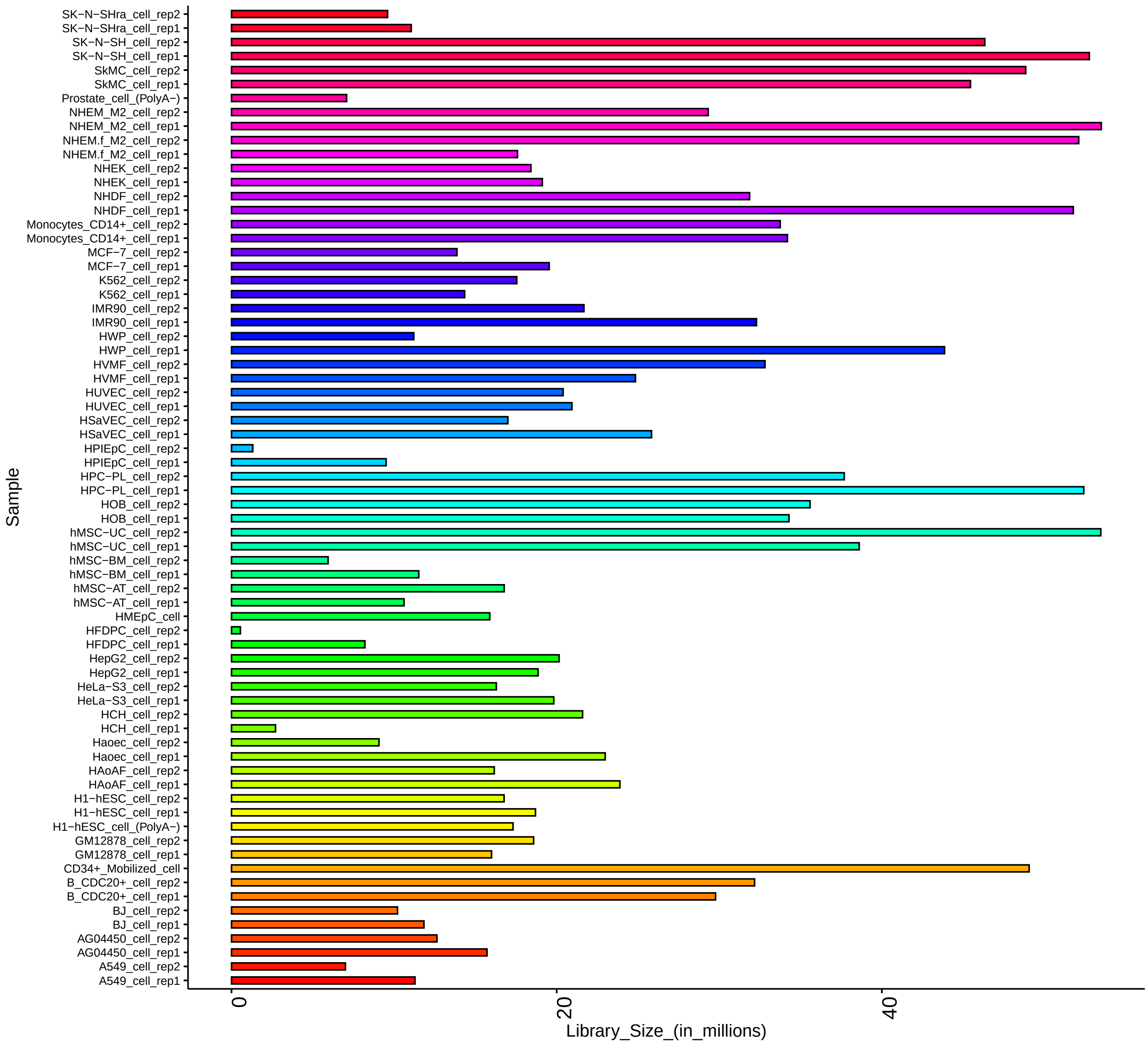

Fig AJ. CAGE library sizes for all different human cell lines available from ENCODE and included in this study.

6.1 Visualisation showing curation of seqArchR result clusters for H.sapiens

In Figures Fig AK, we show the curation of raw clusters (of promoter sequences) from seqArchR result for the CAGE-derived promoters from human cell lines. Note that, here, the clusters are shown ordered by the hierarchical clustering dendrogram (sequence logos on the left). For the sequence logos on the right, the collated clusters are ordered by their IQW. This is the same ordering as in the figures in the main text, including the cluster names.

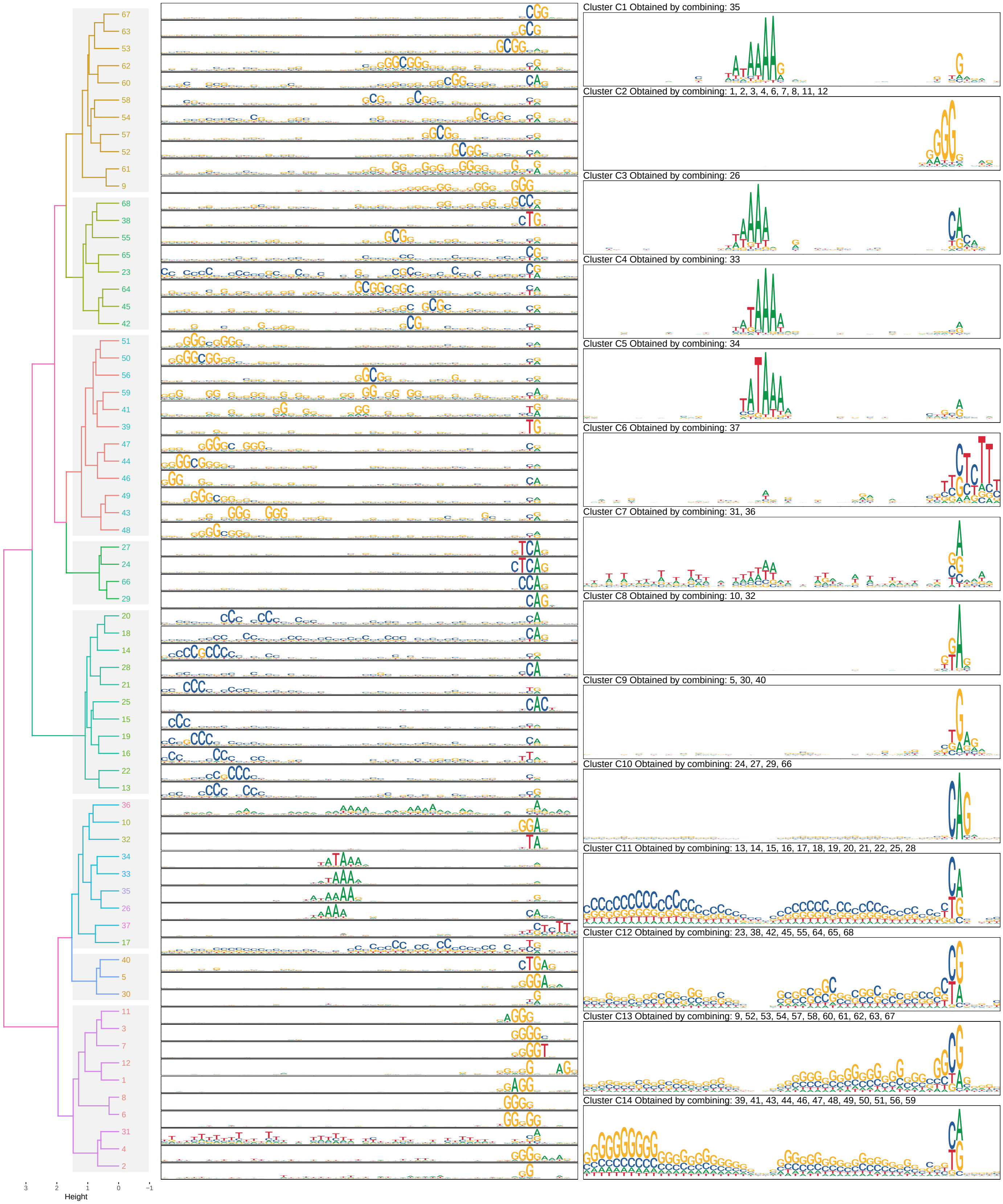

Fig AK. Visualisation of the collation and curation of clusters from seqArchR raw result for H. sapiens

6.2 Comparison of cluster architectures in shorter vs longer downstream flank scenario

To observe the effect of the downstream flanking region on the clusters identified by seqArchR, we processed two sets of core promoter sequences: one with shorter downstream flank (-50, +5 bp) around the dominant CTSS and another with longer downstream flank (-50, +150 bp). Figure Fig AL visualises the sequence logos of promoter architectures per cluster for both cases, shorter flanks on the left, longer flanks on the right. These are separated by a Sankey diagram that shows the movement/exchange of promoter sequences between the two sets of clusters.

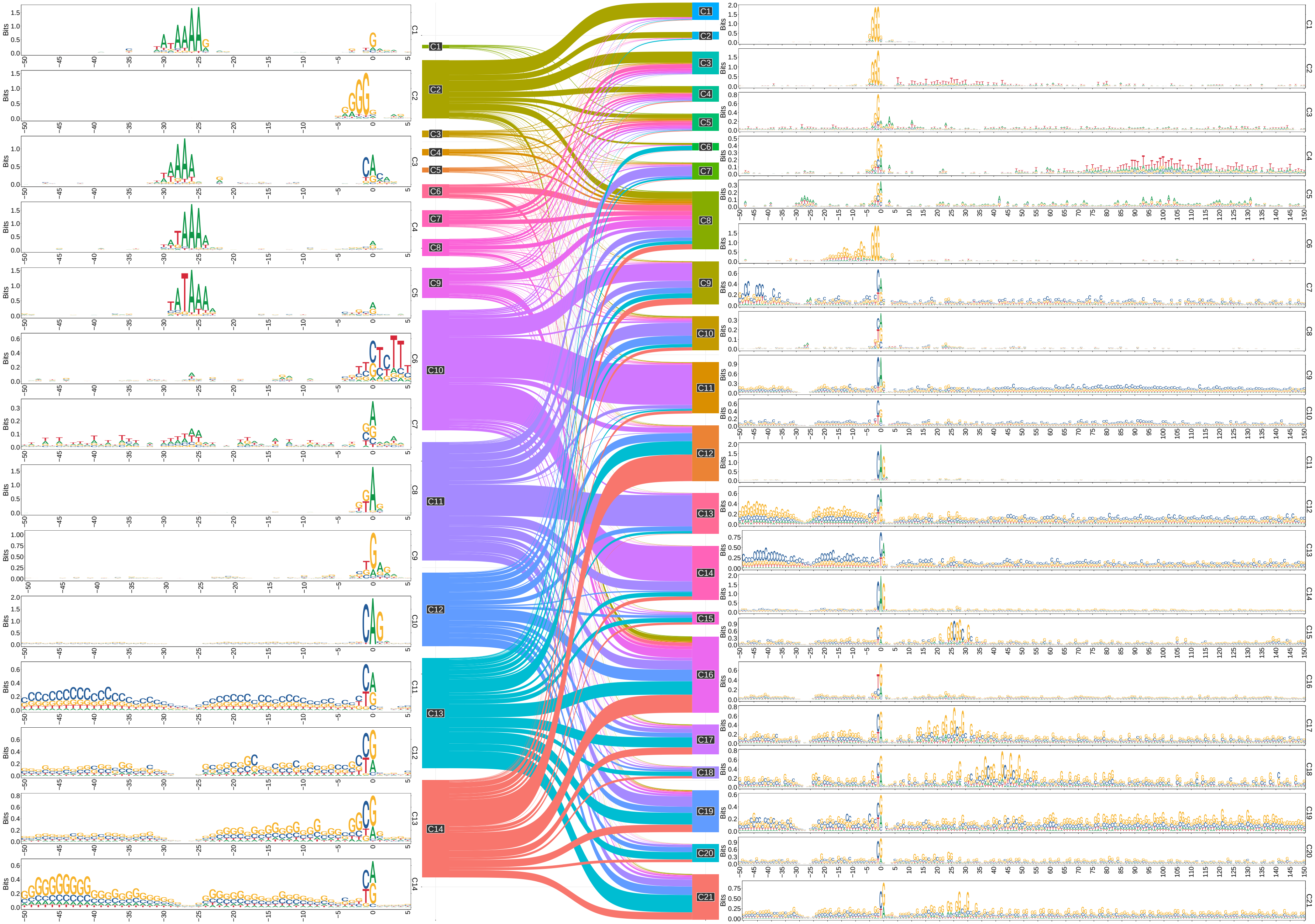

Fig AL. Sankey diagram showing movement of promoter sequences in two clusterings: using flanks of {−50, +5} and {−50, +150} around the dominant TSS.

6.3 Comparison of cluster architectures in two scenarios: with *vs* without initiator sequence

To observe the effect of the initiator sequence (Inr) on the clustering, we excluded the Inr sequence positions from clustering and compared how the clustering of the core promoter sequences changed. In other words, the input promoter sequences were now a concatenation of two spans: $\{-50, -6\}$ and $\{+6, +150\}$, leaving out $\{-5, +5\}$ around the TSS. This is visualised in Figure Fig AM.

The sequence logos on the left are for clusters obtained by retaining/using the Inr sequence $[-5, +5$ around the TSS$]$ when clustering vs excluding it when performing clustering on the right. The Sankey diagram in the middle shows the movement of promoter sequences in the two clusterings. Note that the position labels for the sequence logos on the right go from $\{-50, -6\}$ and $\{+6, +150\}$.

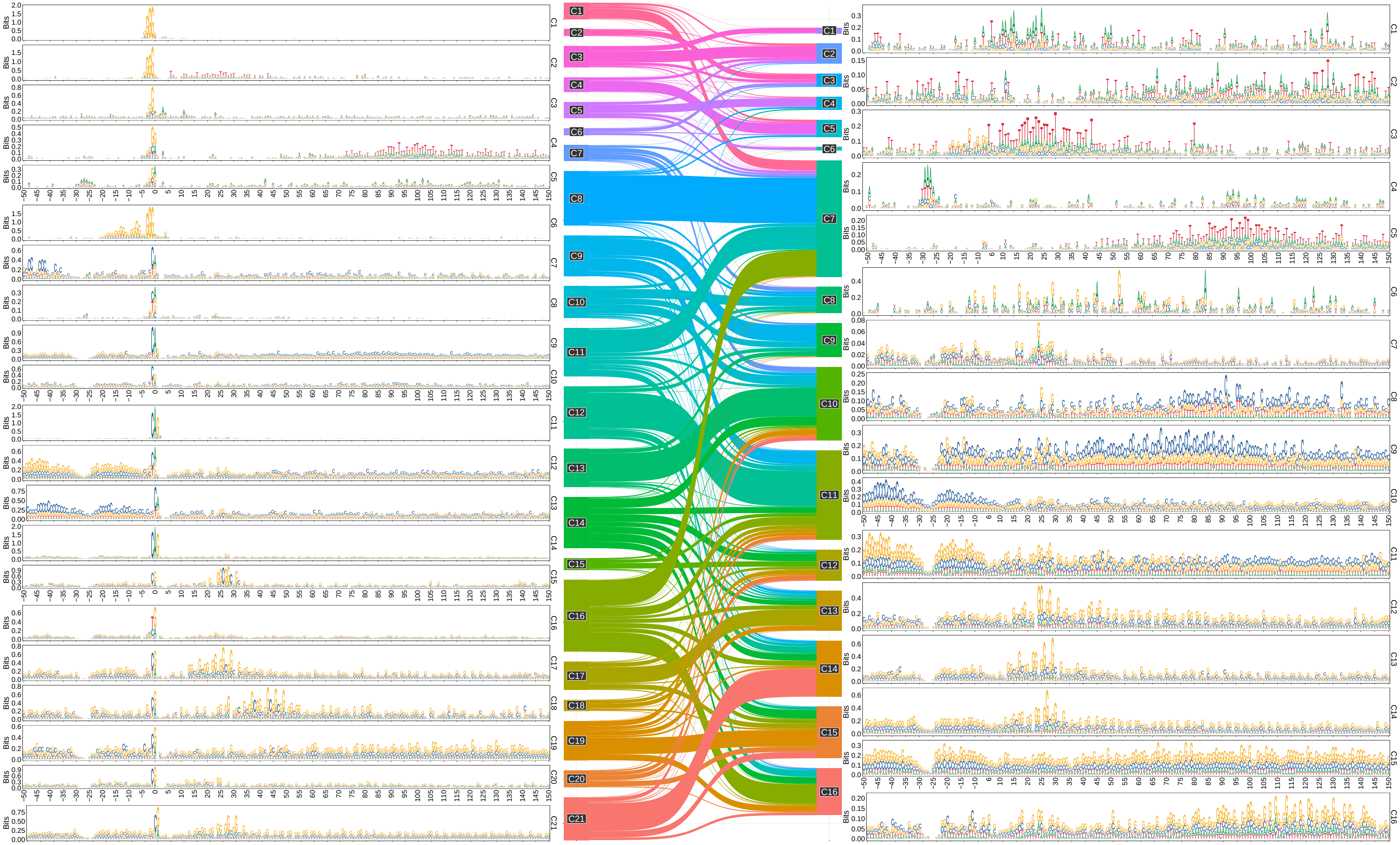

Fig AM. Sankey diagram showing effect of not including Inr sequence on the clustering.

6.4 Diminished tissue-specific signal among the all-pooled CAGE data for H. sapiens

As reported in the main text, we speculate that a lower percentage of tissue-specific genes are identified due to pooling of CAGE data with varying library sizes from various tissues and cell types (as shown in Supplementary Figure Fig AJ).

We verified this by processing, as examples, two single tissue CAGE datasets with the same thresholds as used for the all pooled CAGE data. In this case, we processed the HepG2 (liver) and SkMC (skin melanocytes) CAGE data. We selected these two based on the difference in their library sizes – HepG2 has a moderate library size compared to skin melanocytes which is among the largest (Figure Fig AJ). We annotated the promoters identified in all cases with corresponding genes. This enabled us to identify the genes that are missing from the annotations in the all pooled scenario but are annotated in the individual tissue cases. We refer to these as the 'missed' genes. The histogram below (Figure Fig AN) shows the number of (unique) genes for all deciles of Tau scores (measure of their tissue-specificity; higher values mean highly tissue specific) in three cases (top to bottom): (a) all pooled CAGE data scenario; (b) single tissue CAGE data; and (c) the genes present in single tissue case, but 'missed' in the all pooled case.

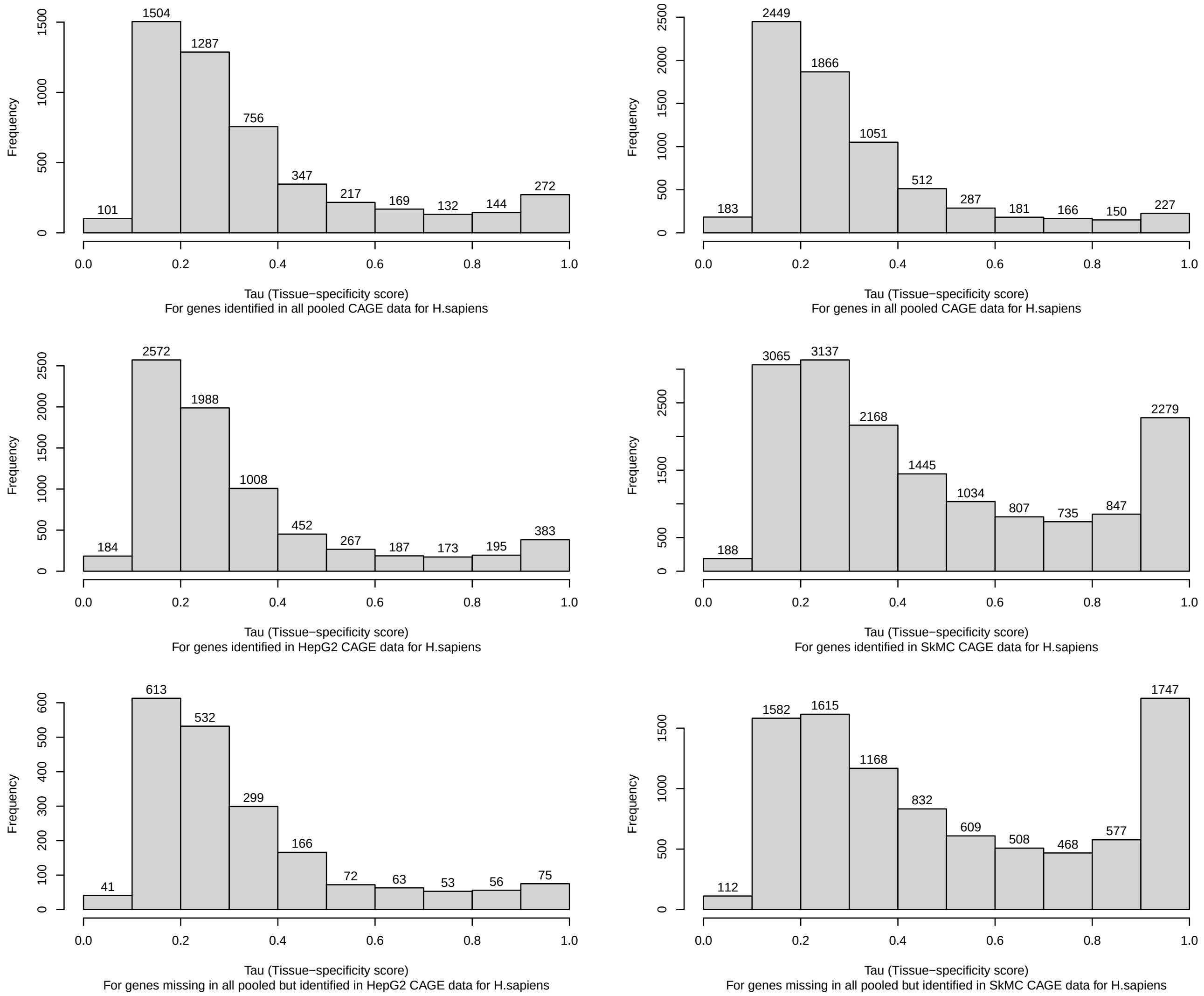

Fig AN. Histograms of number of genes in deciles of Tissue-specificity scores (τ)

References

1. Chen Z-X, Sturgill D, Qu J, Jiang H, Park S, Boley N, et al. Comparative validation of the D. melanogaster modENCODE transcriptome annotation. Genome research. 2014;24:1209--23.
2. Mitra S, Narlikar L. No promoter left behind (NPLB): Learn de novo promoter architectures from genome-wide transcription start sites. Bioinformatics. 2016;32:779--81.
3. Schor IE, Degner JF, Harnett D, Cannavò E, Casale FP, Shim H, et al. Promoter shape varies across populations and affects promoter evolution and expression noise. Nature genetics. 2017;49:550.
4. Grant CE, Bailey TL, Noble WS. FIMO: scanning for occurrences of a given motif. Bioinformatics [Internet]. 2011;27:1017--8. Available from: https://doi.org/10.1093/bioinformatics/btr064
5. Castro-Mondragon JA, Riudavets-Puig R, Rauluseviciute I, Berhanu Lemma R, Turchi L, Blanc-Mathieu R, et al. JASPAR 2022: the 9th release of the open-access database of transcription factor binding profiles. Nucleic Acids Research [Internet]. 2021;50:D165--73. Available from: https://doi.org/10.1093/nar/gkab1113
6. Nepal C, Hadzhiev Y, Previti C, Haberle V, Li N, Takahashi H, et al. Dynamic regulation of the transcription initiation landscape at single nucleotide resolution during vertebrate embryogenesis. Genome research. 2013;23:1938--50.
