# Supplementary material for: Identifying promoter sequence architectures via a chunking-based algorithm using non-negative matrix factorisation
Source: PLoS Comput Biol. 2023 Nov 20;19(11):e1011491. doi: 10.1371/journal.pcbi.1011491 (PMC10695386; doi:10.1371/journal.pcbi.1011491)
Supplement: Attachment — Submitted filename: seqArchR_PCB_reviewer_comments.pdf [file pcbi.1011491.s002.pdf]

Response to Reviewers: PCOMPBIOL-D-23-00587, 'Identifying promoter sequence architectures via a chunking-based algorithm using non-negative matrix factorisation'

Sarvesh Nikumbh[1,2,*] Boris Lenhard[1,2,*]

Contents

1 Introduction **2**

2 Reviews **2**

2.1 Reviewer 1 . . . 2

2.1.1 Obtaining sequence logos from NMF results . . . 2

2.1.2 Sequence logo differences: Fixed 'rigid' motifs *vs* 'short' motifs promoter architectures . . . 2

2.1.3 Selection of factorisation rank and biases . . . 3

2.1.4 Sensitivity of seqArchR to k (factorisation rank/#clusters) . . . 3

2.2 Reviewer 2 . . . 3

2.2.1 Explicitly mentioning post-processing of NMF clusters . . . 4

2.2.2 DPE clearly visible only at 6-8h. Suprising and inconsistent with known knowledge? . . . 4

2.2.3 Introduce the term 'full Inr' before using it . . . 4

2.2.4 Reference for TATA and DPE mutual exclusivity . . . 4

2.2.5 Erroneous Supplementary figure number . . . 5

2.2.6 Textual definition of some variables in Eq. 2 missing . . . 5

2.2.7 More information on C4Z cluster in Zebrafish . . . 5

2.2.8 Are TATA and W-box motifs indeed different? . . . 5

2.2.9 Compare with Dreos et al. PLoS Comput Biol. 2021 . . . 6

2.3 Reviewer 3 . . . 7

2.3.1 Citation required . . . 7

2.3.2 Figure 1 should indicate diversity 'across organisms' . . . 7

2.3.3 Feature vector length . . . 7

2.3.4 Discrepancy in dummy NMF matrix representation in Figure 2 . . . 7

2.3.5 Additional information for cluster A in simulated data experiment . . . 7

2.3.6 Commands to be run for reproducing results . . . 8

2.3.7 Ensuring absence of TATA in some Drosophila clusters . . . 8

2.3.8 Usage of the phrase 'successions of motifs' . . . 8

2.3.9 Hsapiens results: Drowning of TSS-determining motif architecture . . . 8

2.3.10 Hsapiens results: Low percentage of tissue-specific promoter clusters and its verification . . . 9
2.3.11 Confusing usage of the term ‘knockout’ in supplementary text . . . 9
2.3.12 Descriptive results . . . 9
2.3.13 Citation required . . . 9
2.3.14 Explaining variable C_{kj} in Eq. 2 . . . 10
2.3.15 Few cases of language improvement . . . 10

References **10**

[1] Computational Regulatory Genomics, MRC London Institute of Medical Sciences, London, UK
[2] Institute of Clinical Sciences, Faculty of Medicine, Imperial College London, Hammersmith Hospital Campus, London, UK

[*] Correspondence: Sarvesh Nikumbh <s.nikumbh@imperial.ac.uk>, Boris Lenhard <b.lenhard.imperial.ac.uk>

1 Introduction

We thank the three reviewers for their comments, suggestions and questions. In this document we address all the concerns raised by all the reviewers and indicate the corresponding changes in the manuscript.

2 Reviews

2.1 Reviewer 1

Reviewer #1: Review on the article "Identifying promoter sequence architectures via a chunking-based algorithm using non- negative matrix factorisation" by Nikumbh and Lenhard

In this article, the authors present a method to detect promoter motifs using an approach based on non-negative matrix factorization. Promoter sequences are summarized using a one-hot encoding in which all dinucleotides are considered and grouped into several clusters using a multi-step clustering procedure. The authors validate the algorithm using simulated data and show its superior performance with respect to state-of-the-art methods. One advantage of the presented method, seqArchR is the speed of execution. They then apply the method to CAGE data from different organisms and show that they can recover known promoter motifs and related them to specific functional groups. seqArchR can also detect novel motifs, such as in Drosophila, indicating its high sensitivity.

The article is well written, and the results are convincing. The complex algorithm is well described in the methods.

We thank the reviewer for the kind words and for the positive overall assessment of our work!

I have a few questions on some of the results:

2.1.1 Obtaining sequence logos from NMF results

It is not clear how the sequence logos are built from the results of the NMF. This should be described more in detail.

We have added an explanation as a short subsection titled "Visualising promoter architectures as sequence logos from NMF results" under the Methods section explaining this in more detail. See page 29, lines 682-696.

2.1.2 Sequence logo differences: Fixed ‘rigid’ motifs *vs* ‘short’ motifs promoter architectures

Some logos seem to represent fixed motifs (for example C1X, C2X in figure 4), as compared with most other logos which only highlight specific shorter motifs within the promoter sequence. I was wondering why some of the logos have this property. It is probably related to the small number of sequences in the cluster, however Fig S8 seems to

show example of small clusters which do not show this fixed motif logo. Could the authors comment on this, and whether these "rigid" motifs should be removed from the output?

These sequence logos with fixed motifs are observed in Drosophila results, main text, figures 4-6. These are specifically seen for clusters at all three stages (see Table below).

Clusters	Stage
C1-4,6X	2-4h AEL
C2,4-5Y and C8-9Y	6-8h AEL
C1Z and C6-8 Z	10-12h AEL

As discussed in subsection "Detection of recently expanded gene families" in Drosophila results (page 13), these clusters include promoter sequences for histone genes which belong to recently expanded gene families and are thus known to be similar to each other, not having enough time to diverge by accumulating enough neutral mutations. While these clusters indeed have small number of sequences, the logos have maximum height (two bits) at all positions mainly due to them being similar, recently duplicated sequences (i.e., nearly the same sequence throughout in all promoters).

Studies looking to generate a compendium of promoters for an organism and study them further may choose to sequester these from unique promoter sequences to avoid biasing any downstream analyses.

We have added Supplementary Figure 10 showing this percentage of annotations for all seqArchR clusters identified at all stages for D.melanogaster. We also added some text explaining the above: see page 13, lines 299-302.

2.1.3 Selection of factorisation rank and biases

Selection of the factorization rank is a critical aspect in NMF decomposition. The authors present two heuristic methods in the Methods section describing the algorithm. The model selection using bi-cross-validation considers the reconstruction accuracy of the NMF. This sounds to me like the criteria of the Froebenius error measuring the reconstruction error, which generally favors larger values of k. Could the authors comment on possible biases towards higher factorization ranks?

Thank you for raising this point. One of the ways we tackle this bias for higher factorisation rank when using Frobenius error, is using the one-standard error rule with cross-validation where we select the most parsimonious model (factorisation rank k) whose error is no more than one standard error w.r.t. the best model. We have now added this to the algorithm subsection. See page 28, lines 595-597.

2.1.4 Sensitivity of seqArchR to k (factorisation rank/#clusters)

Related to the previous comment, how sensitive is the choice of k in the seqArchR method? How much do the results vary with different values of k?

seqArchR actually requires the specification of boundary values, `k_min` and `k_max`, i.e., the minimal and maximal values of k that will be tested for each sequence chunk. Their default values are 1 and 50 (this is already large enough so that the user does not have to explicitly set it). Selecting the appropriate value of k per chunk of sequences is handled by the model selection procedures described as part of the Algorithm (page 27).

More generally, it is the parameter `bound` (bound on the instability of identified basis vectors) that influences the identification of clusters. With a lenient bound value (10^{-6}), more clusters will be identified in comparison to a stricter bound value (10^{-8}). Some weaker clusters may not be identified with stricter bound values. This is highlighted in the Algorithm subsection, page 28, lines 587-588.

2.2 Reviewer 2

Reviewer #2: This paper describes a (i) new method to classify DNA sequences of fixed length aligned on an experimentally defined position, and (ii) an application of this new method to promoter sequences of three animal species (aligned on TSS). Overall, this is a paper of high scientific quality, clearly written, and presenting interesting

biological results. To me, the usage of non-negative matrix factorization to find subclasses in DNA sequences is novel, and I consider it a pretty smart choice.

The methods are well described, software is available from git hub, and the input data to the computational workflows have been deposited in Zenodo. The study thus meets high standards of reproducibility.

Thank you for the appreciation!

I have a few requests for minor modifications.

Results Section:

2.2.1 Explicitly mentioning post-processing of NMF clusters

My understanding (based on Methods and Supplementary material) is that the architectures shown by sequence logos in the main manuscript were obtained by manual curation and post-processing of automatically generated architectures returned by SeqArchR (shown in supplementary Figures). This should be explicitly and clearly stated in the Results section.

We have now added text in the Results section mentioning this point and referring to the subsection "Curation of collated clusters" in the Methods section of the paper. See page 8, lines 226-228.

2.2.2 DPE clearly visible only at 6-8h. Suprising and inconsistent with known knowledge?

The DPE element is barely visible in the sequence Logos of Drosophila architectures from 2-4h AEL (Fig. 4) and 10-12 AEL transitions. The only architecture with a clearly visible DPE element (C1Y) comes from transition 6-8h AEL. This is surprising and inconsistent with the notion that DPE promoters are characteristic of developmental genes. Please comment.

This is due to collation of similar clusters with strong or weak DPE signal making the motif weak (barely visible) overall in the final logo. For instance, refer to Figure 4 for 2-4h AEL, and the corresponding Supplementary Figure 6. In this case, the cluster C8X (Figure 4) is obtained by collation of clusters 27, 30, 31, 32 and 33 from the raw seqArchR result (Supp Figure 6). As can be seen, there are clusters with stronger DPE signal (27 and 30) and those with weaker signal (31, 32 and 33). Consequently, the resulting cluster from collating these clusters has a weaker DPE signal.

We apologise that this was not clear before. We have now made the following changes to make this clear: explicitly comment and discuss this in the 'Fruit fly' Results section. See page 12, line 256-258 and Table 2.

2.2.3 Introduce the term 'full Inr' before using it

Page 11: "In C8Z, there are two initiators, the canonical CA and full Inr, their A+1 3nt apart". The concept of a "full Inr" should be introduced before in the text, ideally by sequence motif and literature reference.

We have now provided additional information describing the 'full Inr', and also added references to (Ohler et al. 2002; FitzGerald et al. 2006). See page 13, lines 270-271.

2.2.4 Reference for TATA and DPE mutual exclusivity

Page 11: "TATA and DPE sequence elements are known to mutually exclusively pair with the Inr for architectures characteristic of tissue-specific and developmental genes respectively" If possible, justify this statement by a reference.

The earliest paper in which this was reported was (Burke and Kadonaga 1996). The last sentence of its abstract is "These results suggest that the DPE acts in conjunction with the initiation site sequence to provide a binding site for TFIID in the absence of a TATA box to mediate transcription of TATA-less promoters."

Later papers by the same lab have re-iterated this observation, e.g. (Kutach and Kadonaga 2000) and (Vo Ngoc et al. 2020). The latter paper, which deals with (much rarer) human DPE motifs, states explicitly: "This duality between the human DPR and TATA box suggests that they might have different biological functions and are consistent with the mutually exclusive properties of the DPE and TATA box in Drosophila[7,29−32]."

The original categorisation of Drosophila promoters into classes based on motif content (Ohler 2006) also clearly separates TATA+Inr from Inr+DPE promoters. Based on these clusters, we showed that genes TATA+Inr promoters are associated with terminally differentiated biological function terms, and Inr+DPE with embryonic development and transcriptional regulation (Engström et al. 2007).

We have now added the reference to (Burke and Kadonaga 1996) to our original statement.

Methods:

2.2.5 Erroneous Supplementary figure number

Page 22: "Library sizes of all cell lines that are included are shown in Supplementary Figure 4." Should probably be Supplementary Figure 16.

We thank the reviewer for pointing out this discrepancy. We have rectified it.

2.2.6 Textual definition of some variables in Eq. 2 missing

Page 23: Textually define the variables K, C_jk and C_kj occurring in Equation 2.

We have fixed this by adding these definitions in Methods section, sub-section on Algorithm, point 2. "Here, $C_{K\times K}$ is the cross-correlation matrix of basis vectors (columns of W) from R and R' with j and k are column and row iterators."

Below are some comments and suggestions, which the authors may or may not consider at revision stage. I emphasize that they should feel completely free in their choice.

2.2.7 More information on C4Z cluster in Zebrafish

The high information content Logos are intriguing. I find it interesting that some of these logos correspond to histone promoters and one to genes encoding mir-430. I would be curious to know more about other information rich logos, for instance C4Z from zebrafish.

For the revision, we reprocessed zebrafish promoters at all developmental stages with a different chunk size (5K instead of 1K used before). The cluster C4Z from the previous clustering is now cluster C6Z. This cluster contains set of promoters located on chromosome 22, a majority of these on the forward strand. The genes in this locus all belong to a family NACHT-domain and leucine-rich-repeat-containing (NLR) proteins, involved in innate immunity. In zebrafish lineage, this family has been tandemly expanded into approximately 400 copies on chromosome 22 (see https://royalsocietypublishing.org/doi/full/10.1098/rsob.160009). Since the expansion was relatively recent, the duplicated promoter sequences did not have time to diverge, even in neutrally evolving positions.

2.2.8 Are TATA and W-box motifs indeed different?

I have some doubts about the genuine difference between a TATA-box and W-box motif. Since this analysis requires exact superposition for sequence logo generation, W-box-like motifs obtained in this study may simply represent canonical TATA-boxes shifted by +/-2 bp.

Thank you for raising this point. We originally answered it in (Haberle et al. 2014), where we showed that W-box is indeed a degenerate TATA-like motif, and not a canonical TATA box (see Figure 2b and Extended Data Figure 3 in (Haberle et al. 2014)). We later showed that, in mouse, this W-box motif is bound by TBPL2, an oocyte-specific paralog of TBP (TATA binding protein) (Yu et al. 2020), whose sequence specificity is relaxed relative to TBP (which has a strong preference for canonical TATAW core).

In this manuscript, in order to show in the clusters obtained by seqArchR, we reprocessed the promoters of the three Zebrafish developmental stages reported, with a different chunk size (5000 instead of 1000 sequences) and with stability bound values $10^{-6,-7,-8}$. This enabled us to ensure if there are any sequence clusters with `TATA`-box motifs hidden in the `W`-box motif clusters and if seqArchR can identify them. Indeed, in this setting some more specific clusters are identified in comparison to the results of the previous setting. As can be seen in the updated result figures, some clusters with a `TATA`-box signal are identified. The motif occurrence heatmaps shown in Supplementary Figures

28-30 show that there are no occurrences of TATA-boxes in other clusters (aligned occurrences). Corresponding text in the relevant Results section has also been updated. See page number 15-20.

2.2.9 Compare with Dreos et al. PLoS Comput Biol. 2021

A different method based on probabilistic mixture modelling was used in a recent study with partially overlapping objectives (Dreos et al. PLoS Comput Biol. 2021). The authors may comment on the differences in the methodological approaches and/or results obtained for Drosophila and human promoters. One striking difference concerns the Drosophila DPE promoters. According to this other study, more than one third of Drosophila gene promoters conform to an architecture featuring a full Inr and a clear-cut DPE motif (resembling Drosophila C1Y in this paper). Reasons for this discrepancy could be discussed (different gene subsets, upstream Cage data processing method, architecture discovery algorithm, etc).

Following are some important characteristics of the Dreos et al. approach/method which are different in comparison to seqArchR, and which, we believe, give rise to the discrepancy of the results.

1. Dreos et al.'s probabilistic partitioning approach requires specifying K, the number of motifs, which are scored against the sequences and iteratively updated (Expectation-Maximisation). Here, for Drosophila, the authors use $K = 3$ clusters and identify a cluster with Inr+DPE occurring in ca. 37% sequences.

2. The extended algorithm repeats the above procedure n times, and hierarchically clusters the resulting set of $n \times K$ motifs, retaining only K largest clusters to obtain the K average motifs as the final result. Additionally, over-skewing enables their algorithm to identify classes of skewed sizes. Here again, the authors use $K = 6$ clusters for the same set of input sequences, and for Drosophila, identify 4 out of 6 clusters with variations of the DPE which add up to ca. 9% of the same set of input sequences (see Supplementary Fig S1 of Dreos et al.). These variations observed by Dreos et al. are also observed in our results using seqArchR (see Results section, sub-section on Drosophila, and Supplementary figures 6, 7 and 8 showing curation of raw seqArchR clusters).

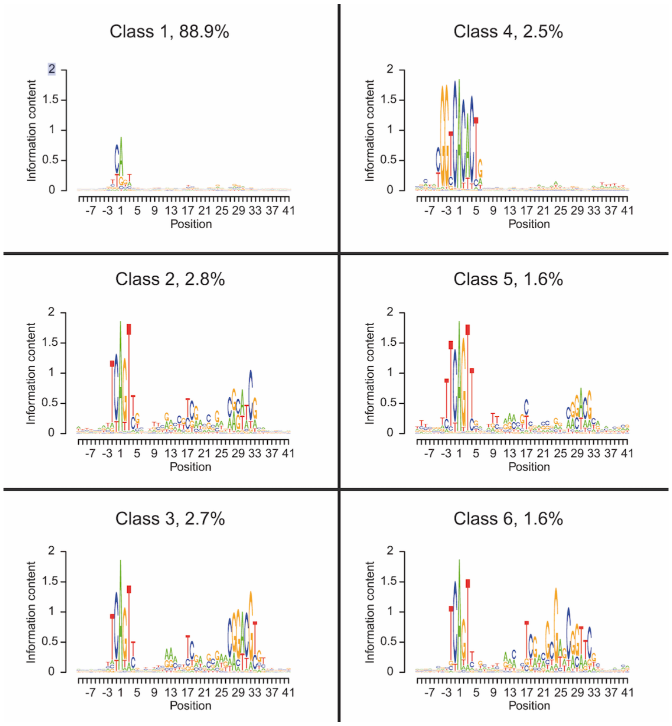

Figure 1: Supplementary Fig S1 of Dreos et al.

3. Processing of CAGE/obtaining promoters: For D.melanogaster, where promoters are obtained from Machibase, Dreos et al. selected, for each gene, the promoters that were validated by the largest number of experiments. This can result in a smaller, high-confidence subset of promoter sequences analysed in their work, and correspondingly, slightly different motifs.

In summary, although the gene promoter sets analysed by Dreos et al. may be different/restrictive, the identified DPE variants and their frequencies are not starkly different. While aims of the Dreos et al. study are different, seqArchR can and has identified motifs missed by Dreos et al. in their work, e.g., the TCT motif in H.sapiens, the TGT motif in Drosophila etc.

2.3 Reviewer 3

Reviewer #3: Nikumbh and Lenhard present a new method for clustering promoter sequences arising from high-resolution experimental data such as CAGE. They use non-negative matrix factorisation for this purpose. The method is timely, given new technologies emerging that give such high-resolution data.

A few issues, which should be clarified:

2.3.1 Citation required

Citation needed on page 3 for "Sharp promoters are associated with tissue-specific genes, while broad promoters are associated with constitutively expressed genes and a subset of tissue restricted ones."

We have first shown this for human and mouse promoters in (Carninci et al. 2006). It has since been shown in Drosophila (Hoskins et al. 2011) and in Amphioxus (Marlétaz et al. 2018). Our most recent review that covers this is (Haberle and Lenhard 2016), which we now refer the readers to after the above statement.

2.3.2 Figure 1 should indicate diversity 'across organisms'

Figure 1: Caption mentions "across organisms", but that is not indicated in the figure.

We have now depicted this information in the figure by addition of silhouettes of different organisms. See Figure 1, page 3.

2.3.3 Feature vector length

Figure 2: If one is using one hot encoding for dinucleotides should the length of the vector not be $4 \times 4 \times (L-1)$*?*

While the vector length will indeed be $4 \times 4 \times (L-1)$, we have padded it with an additional position. In addition to consistency with mono-nucleotide representation, this enables to directly use the NMF basis vectors for downstream visualisation using PWM-like matrices (see section "Visualising promoter architectures as sequence logos from NMF results" in Methods). This does not affect the factorisation, as all the values at the relevant positions in the input matrix and the corresponding positions in both basis matrix and the coefficients matrix are zero.

2.3.4 Discrepancy in dummy NMF matrix representation in Figure 2

The figure, as I understand it, shows an AA at position 2 and at position 4 in the first sequence. Does that not mean the third position should also have an AA? Is the Vpxn matrix a real representation? It should be, ideally.

We have now fixed this discrepancy, and the figure is updated in the manuscript (see page 5 of the manuscript). As for the matrix $V_{p \times n}$, it is indeed real (R_+) in general NMF, but in our case, it has only 0s and 1s representing presence or absence of any feature (dinucleotides) at any given position.

2.3.5 Additional information for cluster A in simulated data experiment

Table 1: Authors should mention the position where the AT period starts in cluster A. The reader can get the wrong impression that the ATs start at arbitrary positions, but occur ever 10 bases.

Thank you, we have now added this information for cluster A in Table 1. See page 5 in the updated manuscript.

2.3.6 Commands to be run for reproducing results

What commands should be run to reproduce the results, in case of both seqArchR and NPLB? These should be stated in the Methods.

We have now added a subsection titled "Reproducibility of results presented in this manuscript" under the Methods section that gives the required information. See page 30, lines 704-716.

2.3.7 Ensuring absence of TATA in some Drosophila clusters

In Fruit fly, authors do not identify the TATA motif in two of the time points. The explanation given may be correct, but one needs to ensure that there really is no TATA motif in those promoters. If you do a scan for the TATA motif is it really not present? Do none of the iterations find it? Would increasing the number of clusters find the TATA motif?

We thank the reviewer for raising this question, and have addressed it as summarised below. A more detailed description is provided in a newly added sub-section, 4.3, to the Supplementary text.

We looked at `TATA` motif pattern heatmaps and FIMO-identified motif PWM matches among seqArchR clusters where no `TATA`-box was already identified. Both, heatmaps and FIMO scans, revealed some cases of unidentified or misclassified `TATA`-box sequences among non–`TATA`-box clusters, i.e., seqArchR clusters with no identified `TATA`-box. Majority of these were found to be in the clusters with a DPE signal. For each stage, those clusters with no `TATA`-box reported were further processed using seqArchR by progressively lenient bound values. We observed that seqArchR could identify roughly 50% of these `TATA`-boxes which were missed earlier. Please refer to sub-section "Ensuring identification of `TATA`-box at all stages in D. melanogaster development" in the Supplementary text for further details.

In summary, the final reported clusters in the main text for all three stages of D. melanogaster have been updated to reflect/accommodate these newly identified `TATA` architecture clusters. The Results section in the main text discusses these new additions (see pages 12-15). The previous results are moved to the Supplementary text, section "seqArchR results for D. melanogaster using stability bound 10^{-8}".

2.3.8 Usage of the phrase 'successions of motifs'

Not sure if "successions of motifs" is common terminology. It appears twice and only in the subheadings. It should be described somewhere, or change it.

We believe "succession" is a reasonably common term for referring to a sequence of changes over a time course -- it is used for e.g. microbial succession (change of composition of microbial species in a culture, biotope or a metagenome) or e.g. succession of transcription factor gene expression in sea urchin gastrulation. We have now added some text explaining this term, "successions of motifs" in the relevant sub-section. See page 15, line 316.

2.3.9 Hsapiens results: Drowning of TSS-determining motif architecture

On page 19, the authors state "To try to prevent drowning of this TSS-determining motif architecture. . . " This needs to be qualified by a figure with the original results of using the full sequence length. Furthermore, instead of going only after the TATA motif, why not also look at the downstream regions where the claim is that there exists "dominant influence of the downstream flanking sequence"? More explanation/analysis is necessary here.

We apologise that we did not clearly state earlier that the original results for *H. sapiens* using the full sequence length, $\{-50, +150\}$ bp, is shown in Supplementary figure 29 (sequence logos on the right hand side of the sankey diagram in the figure). As noted, these sequence logos show that with the same bound value of 10^{-6}, the `TATA` signal identified is much weaker (than the canonical `TATA`) when using a longer downstream flank than shorter.

By '. . . dominant influence of the downstream flanking sequence', we mean its influence on identifying clusters. By reducing/not including the downstream flank, we see that clusters with a relatively stronger `TATA` signal are identified (refer main text, H. sapiens results, Figure 12). We modified the main text to make this clear.

2.3.10 Hsapiens results: Low percentage of tissue-specific promoter clusters and its verification

- *"We speculate that the reason for this low percentage could be due to the way the set of promoters was obtained: by pooling CAGE signal from many different tissue and cell types where signal from tissue-specific genes is diluted, unlike that of broadly expressed genes, bringing a higher proportion of the later under the 1TPM threshold."*

(1) *This can be verified. If you take a single tissue, and use CAGE only from that, do you see more tissue-specific signals?*

(2) *In the very least, does one lose out on tissue-specific genes when this combined approach is taken to identify promoters?*

We have added a section in the supplementary text verifying this speculation. In summary, we compared two single tissue CAGE datasets, as examples, with the all pooled CAGE data by processing it using the same thresholds. Then we looked at the genes not identified in the all pooled case but identified in the individual single tissue case. As seen in Supplementary Figure 40 that shows the tissue-specificity scores (τ scores) of these 'missed' genes, many of these are highly tissue-specific ($\tau > 0.5$).

2.3.11 Confusing usage of the term 'knockout' in supplementary text

- *Supplementary Figure 12 refered to on page 19 is for zebrafish. It should probably be Supplementary Figure 19. That supplementary section contains the word "knockout". The paragraph in the main paper only talks about "with and without the initiator sequence". What exactly was done?*

Apologies for the confusing terminology here. We have improved the text in the Supplementary data (page 24, supplementary section 6.3) to match the terminology used in the main text. Essentially, we removed the term "knock-out", the cause of the confusion, and replaced it with "with and without initiator sequence". To clarify, there was no experimental knock-out of the initiator sequence performed, but it was only excluded computationally from the clustering process. This was achieved by using a shortened promoter sequence that was a concatenation of two spans, $\{-50, -6\}$ nt and $\{+6, +150\}$ nt, and leaving out $\{-5, +5\}$ nt around the TSS, instead of the full $\{-50, +150\}$ nt region around the TSS.

2.3.12 Descriptive results

- *The results are very descriptive in nature, can any significance be associated with them?*

The aims of this paper are two-fold.

1. To identify the often under-appreciated diversity in promoter sequence architectures across organisms, and, even within organisms, across developmental stages. This also reinforces the argument that using only a few known motifs to identify and study genome-wide promoters would be hugely insufficient to understand the complete picture.
2. To showcase seqArchR's capabilities to assist in generating compendiums of genome-wide promoter architectures that enable studying this diversity. seqArchR can also extend this capability to less-studied, non-model organisms.

2.3.13 Citation required

- *Citation needed for "It is known that dinucleotide profiles of DNA sequences hold more information than mononucleotide profiles" on page 23.*

This has been fixed by adding two relevant references, namely (Mathelier and Wasserman (2013)) and (Siddharthan (2010)). See line 544, page 27.

2.3.14 Explaining variable C_{kj} in Eq. 2

What is Ckj in equation 2?

DONE This is also raised by Reviewer 2 — thus, is already addressed above.

2.3.15 Few cases of language improvement

A few places where language can be improved:

1. *Page 2:"happens by assembling the pre-initiation complex" -> "happens by assembly of the pre-initiation complex"*
2. *Page 3: authors say "as is traditional, has a limitation" but go on to list more than one.*
3. *Page 4: "suits well" -> "is well-suited"*
4. *Page 21: "fatorisation"*

Thanks for pointing out these problems, as well as for the suggestions, all of which have been accepted.

References

Burke, Thomas W, and James T Kadonaga. 1996. "Drosophila TFIID Binds to a Conserved Downstream Basal Promoter Element That Is Present in Many TATA-Box-Deficient Promoters." *Genes & Development* 10 (6): 711--24.

Carninci, Piero, Albin Sandelin, Boris Lenhard, Shintaro Katayama, Kazuro Shimokawa, Jasmina Ponjavic, Colin AM Semple, et al. 2006. "Genome-Wide Analysis of Mammalian Promoter Architecture and Evolution." *Nature Genetics* 38 (6): 626--35.

Engström, Pär G, Shannan J Ho Sui, Øyvind Drivenes, Thomas S Becker, and Boris Lenhard. 2007. "Genomic Regulatory Blocks Underlie Extensive Microsynteny Conservation in Insects." *Genome Research* 17 (12): 1898--908.

FitzGerald, Peter C, David Sturgill, Andrey Shyakhtenko, Brian Oliver, and Charles Vinson. 2006. "Comparative Genomics of Drosophila and Human Core Promoters." *Genome Biology* 7 (7): 1--22.

Haberle, Vanja, and Boris Lenhard. 2016. "Promoter Architectures and Developmental Gene Regulation." In *Seminars in Cell & Developmental Biology*, 57:11--23. Elsevier.

Haberle, Vanja, Nan Li, Yavor Hadzhiev, Charles Plessy, Christopher Previti, Chirag Nepal, Jochen Gehrig, et al. 2014. "Two Independent Transcription Initiation Codes Overlap on Vertebrate Core Promoters." *Nature* 507 (7492): 381--85.

Hoskins, Roger A, Jane M Landolin, James B Brown, Jeremy E Sandler, Hazuki Takahashi, Timo Lassmann, Charles Yu, et al. 2011. "Genome-Wide Analysis of Promoter Architecture in Drosophila Melanogaster." *Genome Research* 21 (2): 182--92.

Kutach, Alan K, and James T Kadonaga. 2000. "The Downstream Promoter Element DPE Appears to Be as Widely Used as the TATA Box in Drosophila Core Promoters." *Molecular and Cellular Biology* 20 (13): 4754--64.

Marlétaz, Ferdinand, Panos N Firbas, Ignacio Maeso, Juan J Tena, Ozren Bogdanovic, Malcolm Perry, Christopher DR Wyatt, et al. 2018. "Amphioxus Functional Genomics and the Origins of Vertebrate Gene Regulation." *Nature* 564 (7734): 64--70.

Mathelier, Anthony, and Wyeth W Wasserman. 2013. "The Next Generation of Transcription Factor Binding Site Prediction." *PLoS Computational Biology* 9 (9): e1003214.

Ohler, Uwe. 2006. "Identification of core promoter modules in Drosophila and their application in accurate transcription start site prediction." *Nucleic Acids Research* 34 (20): 5943--50. https://doi.org/10.1093/nar/gkl608.

Ohler, Uwe, Guo-chun Liao, Heinrich Niemann, and Gerald M Rubin. 2002. "Computational Analysis of Core Promoters in the Drosophila Genome." *Genome Biology* 3 (12): 1--12.

Siddharthan, Rahul. 2010. "Dinucleotide Weight Matrices for Predicting Transcription Factor Binding Sites: Generalizing the Position Weight Matrix." *PloS One* 5 (3): e9722.

Vo Ngoc, Long, Cassidy Yunjing Huang, California Jack Cassidy, Claudia Medrano, and James T Kadonaga. 2020. “Identification of the Human DPR Core Promoter Element Using Machine Learning.” *Nature* 585 (7825): 459--63.

Yu, Changwei, Nevena Cvetesic, Vincent Hisler, Kapil Gupta, Tao Ye, Emese Gazdag, Luc Negroni, et al. 2020. “TBPL2/TFIIA Complex Establishes the Maternal Transcriptome Through Oocyte-Specific Promoter Usage.” *Nature Communications* 11 (1): 6439.
